# Cell growth dynamics in two types of apical meristems in fern gametophytes

**DOI:** 10.1111/tpj.15784

**Published:** 2022-05-12

**Authors:** Xiao Wu, An Yan, Xi Yang, Jo Ann Banks, Shaoling Zhang, Yun Zhou

**Affiliations:** ^1^ Department of Botany and Plant Pathology Purdue University West Lafayette Indiana 47907 USA; ^2^ Purdue Center for Plant Biology Purdue University West Lafayette Indiana 47907 USA; ^3^ State Key Laboratory of Crop Genetics and Germplasm Enhancement, Center of Pear Engineering Technology Research Nanjing Agricultural University Nanjing Jiangsu 210095 China; ^4^ Division of Biology and Biological Engineering California Institute of Technology Pasadena California 91125 USA; ^5^ Howard Hughes Medical Institute California Institute of Technology Pasadena California 91125 USA

**Keywords:** cell division, ferns, apical cell, apical meristem, gametophytes, cell size, *Woodsia obtusa*

## Abstract

In contrast to seed plants, the gametophytes of seed‐free plants develop pluripotent meristems, which promote and sustain their independent growth and development. To date, the cellular basis of meristem development in gametophytes of seed‐free ferns remains largely unknown. In this study, we used *Woodsia obtusa*, the blunt‐lobe cliff fern, to quantitatively determine cell growth dynamics in two different types of apical meristems – the apical initial centered meristem and the multicellular apical meristem in gametophytes. Through confocal time‐lapse live imaging and computational image analysis and quantification, we determined unique patterns of cell division and growth that sustain or terminate apical initials, dictate the transition from apical initials to multicellular apical meristems, and drive proliferation of apical meristems in ferns. Quantitative results showed that small cells correlated to active cell division in fern gametophytes. The marginal cells of multicellular apical meristems in fern gametophytes undergo division in both anticlinal and periclinal orientations, not only increasing cell numbers but also playing a dominant role in increasing cell layers during gametophyte development. All these findings provide insights into the function and regulation of meristems in gametophytes of seed‐free vascular plants, suggesting both conserved and diversified mechanisms underlying meristem cell proliferation across land plants.

## Significance Statement

In contrast to seed plants, seed‐free ferns develop meristems in their gametophytes. This work quantitatively uncovers the cellular basis of the initiation and proliferation of two different meristems in fern gametophytes.

## INTRODUCTION

In land plants, alternation of generations occurs between sexual gametophytes and asexual sporophytes (Frank & Scanlon, 2015; Horst et al., 2016; Plackett et al., 2015). Sporophytes of both seed plants and seed‐free vascular plants develop shoot apical meristems (SAMs) and root apical meristems, to drive organ formation and shape the architecture of shoots and roots, respectively (Harrison, 2017; Harrison & Morris, 2018; Heidstra & Sabatini, 2014; Philipson, 1990; Plackett et al., 2015). In contrast, the gametophytes of seed‐free ferns are independent of their sporophytes, and they initiate and maintain pluripotent meristems to sustain independent growth and cell proliferation (Nayar & Kaur, 1971; Plackett et al., 2015, 2018; Rensing, 2017). Compared to the well‐characterized meristems in the sporophytes of seed plants, especially in the model flowering plant Arabidopsis (Greb & Lohmann, 2016; Han et al., 2020; Meyerowitz, 1997; Tsukaya, 2021; Zhou et al., 2015, 2018), studies in ferns just started shedding light on meristem development in fern gametophytes (Plackett et al., 2015, 2018; Wu et al., 2021). Previous work showed that gametophytes of different fern species develop three types of meristems, including the apical initial (and its immediate derivatives), which is also called the apical cell‐based meristem, the multicellular meristem, and the marginal meristem (Banks, 1999; Bartz & Gola, 2018; Conway & Di Stilio, 2020; Imaichi, 2013; Nayar & Kaur, 1971; Takahashi et al., 2009, 2012, 2015; Wu et al., 2021). Among them, the marginal meristem is well characterized in *Colysis decurrens* (Takahashi et al., 2009; Imaichi, 2013) and it is also referred to as the multicellular meristem in several other species (Atkinson & Stokey, 1964; Bartz & Gola, 2018), whereas the apical cell (apical initial)‐based meristem and the multicellular meristem are distinct (Banks, 1999; Imaichi, 2013; Nayar & Kaur, 1971). An apical initial usually contains a single cell located at the apex of the gametophyte, and it is characterized by a unique wedge‐shaped morphology (Imaichi, 2013; Nayar & Kaur, 1971). It has been proposed that the apical initial can continuously divide and drive cell proliferation during fern gametophyte development (Nayar & Kaur, 1971). In contrast to the apical initial, a multicellular meristem is composed of a group of adjacent rectangular cells at either the apex or the lateral side of a gametophyte (Imaichi, 2013; Nayar & Kaur, 1971). The multicellular meristem also promotes cell proliferation and drives gametophyte development, and it eventually forms a concave meristem notch (Banks, 1999; Imaichi, 2013; Nayar & Kaur, 1971). In a schizaeoid fern, *Lygodium japonicum* (Lygodiaceae), the apical initial cell shows the typical wedge‐shaped morphology and it drives cell proliferation in gametophytes from the anterior side for a limited time (Takahashi et al., 2015). Then, the multicellular apical meristem is formed, continuously sustaining prothallus proliferation (Takahashi et al., 2015). In the widely studied model fern *Ceratopteris richardii* (Banks, 1999; Bui et al., 2015; Chatterjee & Roux, 2000; Cooke et al., 1995; Eberle et al., 1995; Geng et al., 2021; Hickok et al., 1987, 1995; Marchant et al., 2019; Plackett et al., 2015, 2018; Wu et al., 2021), gametophyte development is predominately driven by one type of multicellular meristem specifically initiated at one lateral side, also called the lateral meristem, the marginal meristem, or the notch meristem (Banks, 1999; Banks et al., 1993; Bartz & Gola, 2018; Conway & Di Stilio, 2020). In contrast, the presence of an apical initial in *C. richardii* gametophytes is very transient and its activity was hardly observed (Bartz & Gola, 2018). The initiation and maintenance of the multicellular meristem in *C. richardii* drives the active proliferation from the lateral side instead of the apex of a prothallus (Banks, 1999; Banks et al., 1993; Bartz & Gola, 2018; Conway & Di Stilio, 2020). A recent study showed that an apical initial cell and a multicellular marginal meristem are present at the same time but in different regions of a *Pteris vittata* gametophyte (Wu et al., 2021). The co‐existence of these two types of meristems in *P. vittata* drives prothallus proliferation in two different directions, eventually leading to variable morphology of the gametophytes in this species (Wu et al., 2021). Despite this progress in these two related ferns (*C. richardii* and *P. vittata*) from the family Pteridaceae, the cell growth dynamics within these two types of gametophyte meristems in ferns, especially across different families, still remains largely unclear. To address this question, we used *Woodsia obtusa* – the blunt‐lobe cliff fern – as a research system in this study for several major reasons. First, the previously characterized *L. japonicum* (Lygodiaceae) belongs to the order Schizaeales, relatively basal in phylogeny (Pryer et al., 2004; Takahashi et al., 2015). In the order Polypodiales, distantly related to *C. richardii* (Pteridaceae) and *P. vittata* (Pteridaceae), *W. obtusa* (Woodsiaceae) belongs to the suborder Aspleniineae and represents an important but yet underexplored branch in phylogeny (PPG, I., 2016). Therefore, quantitative analyses of meristem cell behaviors in *W. obtusa* gametophytes will provide a more comprehensive view of meristem evolution across different fern families. Second, we found that the *W. obtusa* gametophyte does not have any multicellular marginal meristem but subsequently develops the active apical initial and the multicellular apical meristem within the same region at different developmental stages. As a flat sheet of cells, the *W. obtusa* gametophyte is ideal for the non‐invasive time‐lapse confocal imaging and two‐dimensional (2D) imaging analysis pipeline that we have established (Wu et al., 2021). Therefore, *W. obtusa* serves as an efficient research system to explore the transition between these two meristem identities at single‐cell resolution. Third, we found that *W*. *obtusa* gametophytes develop multiple trichomes, which were independent of apical initials and multicellular apical meristems. Quantitative imaging in *W. obtusa* will not only reveal the cellular basis of trichome development in gametophytes, but also uncover the potential communication and interaction between undifferentiated meristems and differentiated trichomes in fern gametophytes.

Through non‐invasive time‐lapse confocal imaging and computational image analysis, we quantitatively determined the patterns of cell division and growth that were directly associated with the initiation and proliferation of apical meristems and trichomes in *W*. *obtusa* gametophytes. Our results provide insights into cell proliferation in fern gametophytes and suggest both conserved and diversified mechanisms underlying apical meristem development in land plants.

## RESULTS

### *Woodsia obtusa* gametophytes develop apical initials, multicellular apical meristems, and trichomes

From the optical microscope photographs, we found that a *W. obtusa* gametophyte developed one flat sheet of cells at 24 days after inoculation (DAI) (Figure S1a,b). At 38 DAI, the *W. obtusa* gametophyte developed a multicellular apical meristem with a shallow notch at the apical side (Figure S1c,d). It also developed several trichomes at the surface; however, it was lacking any wedge‐shaped apical initial at 38 DAI (Figure S1c,d). To capture the morphology of *W. obtusa* gametophytes in high resolution, we grew the *W. obtusa* spores on solidified growth medium and imaged different individual gametophytes right after their germination using laser scanning confocal microscopy (Figure S2). The single spore cell emerged from the spore coat at 7–8 DAI and it continued to expand in area and divided into three or four cells (Figure S2a). The wedge‐shaped apical initial (marked by the star) was distinguishable in the gametophytes at 16–22 DAI (Figure S2b–f). These results suggested that at early developmental stages (16–22 DAI), an apical initial likely plays a role in the proliferation of *W. obtusa* gametophytes (Figure S2). At 17 DAI, one trichome emerged at the top of the gametophyte (Figure S2c), and more trichomes initiated and expanded as the gametophytes developed (Figure S2c–f), suggesting the initiation of trichomes in *W. obtusa* gametophytes is an early event, prior to the initiation of multicellular apical meristems. All these optical microscope photographs (Figure S1) and confocal snapshots (Figure S2) suggested that *W. obtusa* develops both apical cells and multicellular apical meristems and likely undergoes a transition from an apical cell‐based meristem to a multicellular apical meristem during development. These findings informed time‐lapse experiments designed to determine the cellular basis of the initiation and proliferation of these two types of apical meristems.

### Cell growth dynamics in the apical initial and its immediate progenies in gametophytes

To determine the dynamic patterns of cell proliferation in the apical meristems of *W. obtusa* gametophytes, we performed confocal time‐lapse imaging to follow the division and growth of each cell in living *W. obtusa* gametophytes at different time points (Figures 1, 2, 3, 4, 5, 6, 7; Figures S3–S12). We then performed computational image analysis to segment cells from the confocal images, quantify the area of each segmented cell, and analyze the patterns and activity of cell division (Figures  1, 2, 3, 4, 5, 6; Figures S3–S12, Tables S1 and S2).

**Figure 1:**
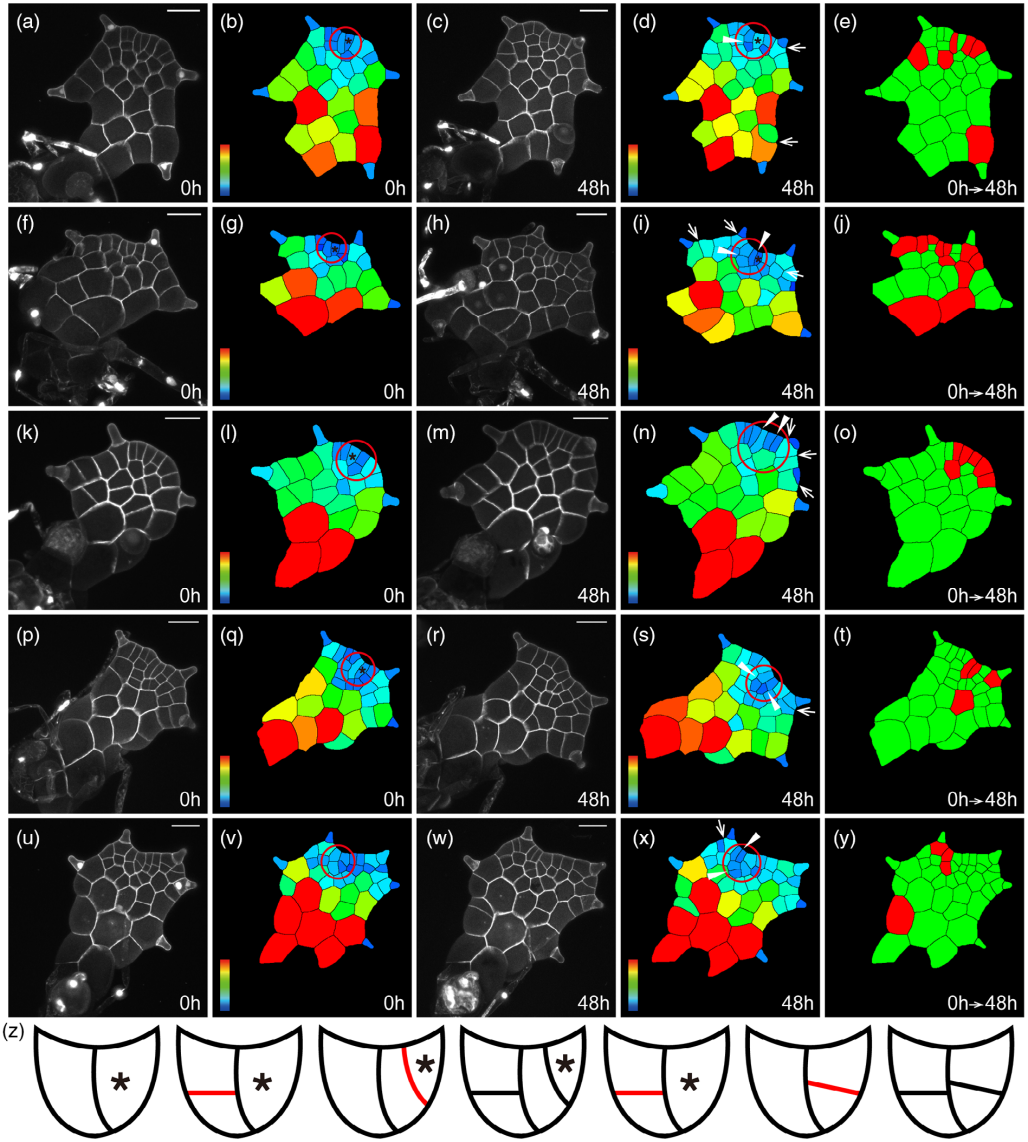
The distinct patterns of cell division in the apical initials. (a–y) Five *Woodsia obtusa* gametophytes (a–e, f–j, k–o, p–t, u–y) were stained and live‐imaged through laser scanning confocal microscopy at 0 h (a, f, k, p, u) and 48 h (c, h, m, r, w). (b, d, g, i, l, n, q, s, v, x) Computational segmentation and cell size quantification of confocal images in (a, c, f, h, k, m, p, r, u, w), respectively. (e, j, o, t, y) Cell division in the gametophytes in (a, f, k, p, u), with the cells that divided during the analyzed period (48 h) shown in red and the cells that did not divide during the same time period shown in green. (a, f, p, u) The gametophytes at 23 days after inoculation (DAI). (k) The gametophyte at 25 DAI. (z) Diagrams and illustration of cell division patterns during the proliferation and termination of apical initials, with newly formed cell wall shown in red. At least three independent biological replicates showed similar patterns of cell division summarized in (z). (a, c, f, h, k, m, p, r, u, w) Gray: propidium iodide (PI) stain; scale bars: 50 μm. Color bars in (b, d, g, i, l, n, q, s, v, x) indicate the quantified area of each segmented cell, with the scale ranging from blue (0) to red (≥2000 μm^2^). In (b, d, g, i, l, n, q, s, v, x), red circles indicate the conserved cell packets that contain apical initials and stars indicate the wedge‐shaped apical initials. In (b, d, g, i, l, n, q, s, v, x), white triangles indicate cell division in the cell packets of apical initials and white arrows indicate cell division associated with trichome development. [Colour figure can be viewed at wileyonlinelibrary.com]

**Figure 2:**
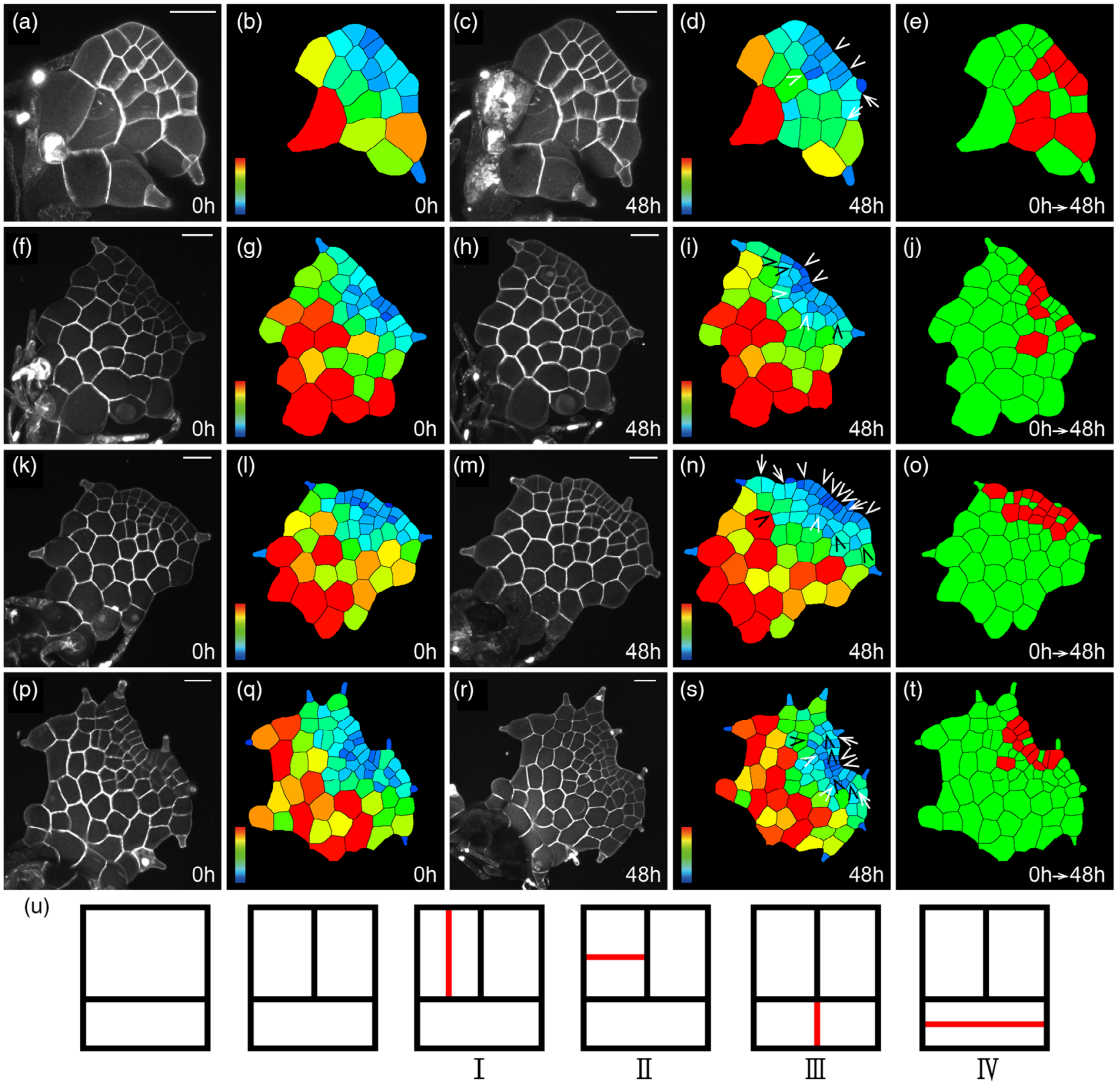
The patterns of cell division during the initiation of multicellular apical meristems in gametophytes. (a–t) Four *Woodsia obtusa* gametophytes (a–e, f–j, k–o, p–t) were stained and live‐imaged through laser scanning confocal microscopy at 0 h (a, f, k, p) and 48 h (c, h, m, r). (b, d, g, i, l, n, q, s) The computational segmentation and cell size quantification of confocal images in (a, c, f, h, k, m, p, r), respectively. (e, j, o, t) Cell division in the gametophytes in (a, f, k, p), with the cells that divided during the analyzed period (48 h) shown in red and the cells that did not divide during the same time period shown in green. (a) The gametophyte at 23 days after inoculation (DAI). (f, k, p) The gametophytes at 28 DAI. (u) Diagrams and illustration of cell division patterns during the initiation of multicellular apical meristems, with newly formed cell wall in red. I, II, III, and IV indicate four different patterns of cell division in the three‐celled packet from multicellular meristems, which are also related to the quantification results in Figure 5a,b. At least three independent biological replicates showed similar patterns of cell division during the initiation of multicellular apical meristems. (a, c, f, h, k, m, p, r) Gray: propidium iodide (PI) stain; scale bar: 50 μm. Color bars in (b, d, g, i, l, n, q, s) indicate the quantified area of each segmented cell, with the scale ranging from blue (0) to red (≥2000 μm^2^). In (b, d, g, i, l, n, q, s), white ‘V’ indicates anticlinal division in multicellular meristems, black ‘V’ indicates periclinal division in multicellular meristems, and white arrows indicate cell division associated with trichome development. [Colour figure can be viewed at wileyonlinelibrary.com]

**Figure 3:**
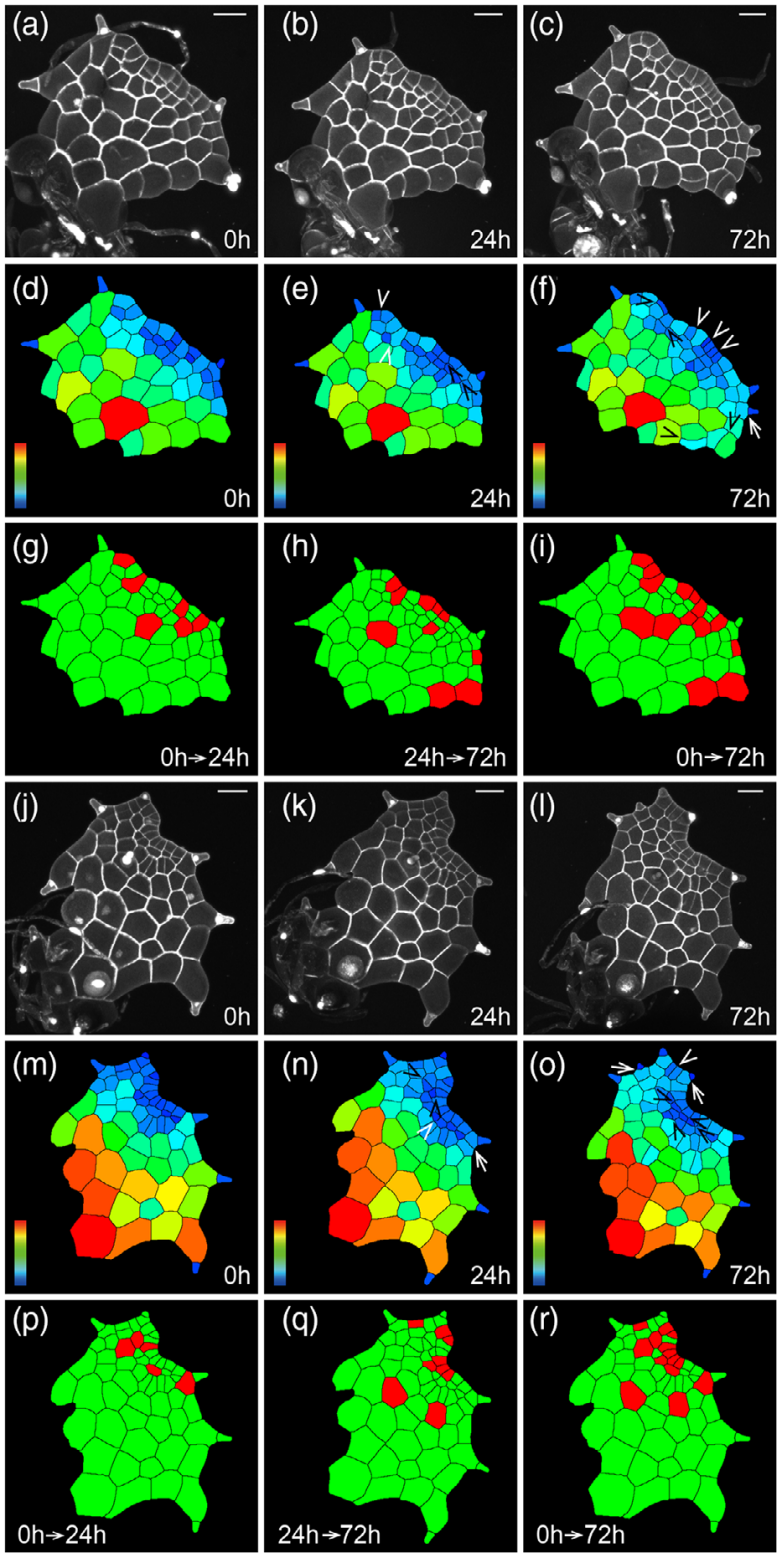
The patterns of cell division during the proliferation of multicellular apical meristems in gametophytes. (a–r) Two *Woodsia obtusa* gametophytes (a–i, j–r) were stained and live‐imaged through laser scanning confocal microscopy at 0 h (a, j), 24 h (b, k), and 72 h (c, l). (d, e, f, m, n, o) Computational segmentation and cell size quantification of confocal images in (a, b, c, j, k, l), respectively. (g, i, p, r) Cell division in the gametophytes in (a, j) over 24 h (g, p) and over 72 h (i, r). (h, q) Cell division in the gametophytes in (b, k) over 48 h. The cells that divided during the analyzed period are shown in red and the cells that did not divide during the analyzed period are shown in green. (a, j) Gametophytes at 29 days after inoculation (DAI). At least three independent biological replicates showed similar patterns of cell division during the proliferation of multicellular apical meristems. (a, b, c, j, k, l) Gray: propidium iodide (PI) stain; scale bar: 50 μm. Color bars in (d, e, f, m, n, o) indicate the quantified area of each segmented cell, with the scale ranging from blue (0) to red (≥3000 μm^2^). In (d, e, f, m, n, o), white ‘V’ indicates anticlinal division in multicellular meristems, black ‘V’ indicates periclinal division in multicellular meristems, and white arrows indicate cell division associated with trichome development. [Colour figure can be viewed at wileyonlinelibrary.com]

**Figure 4:**
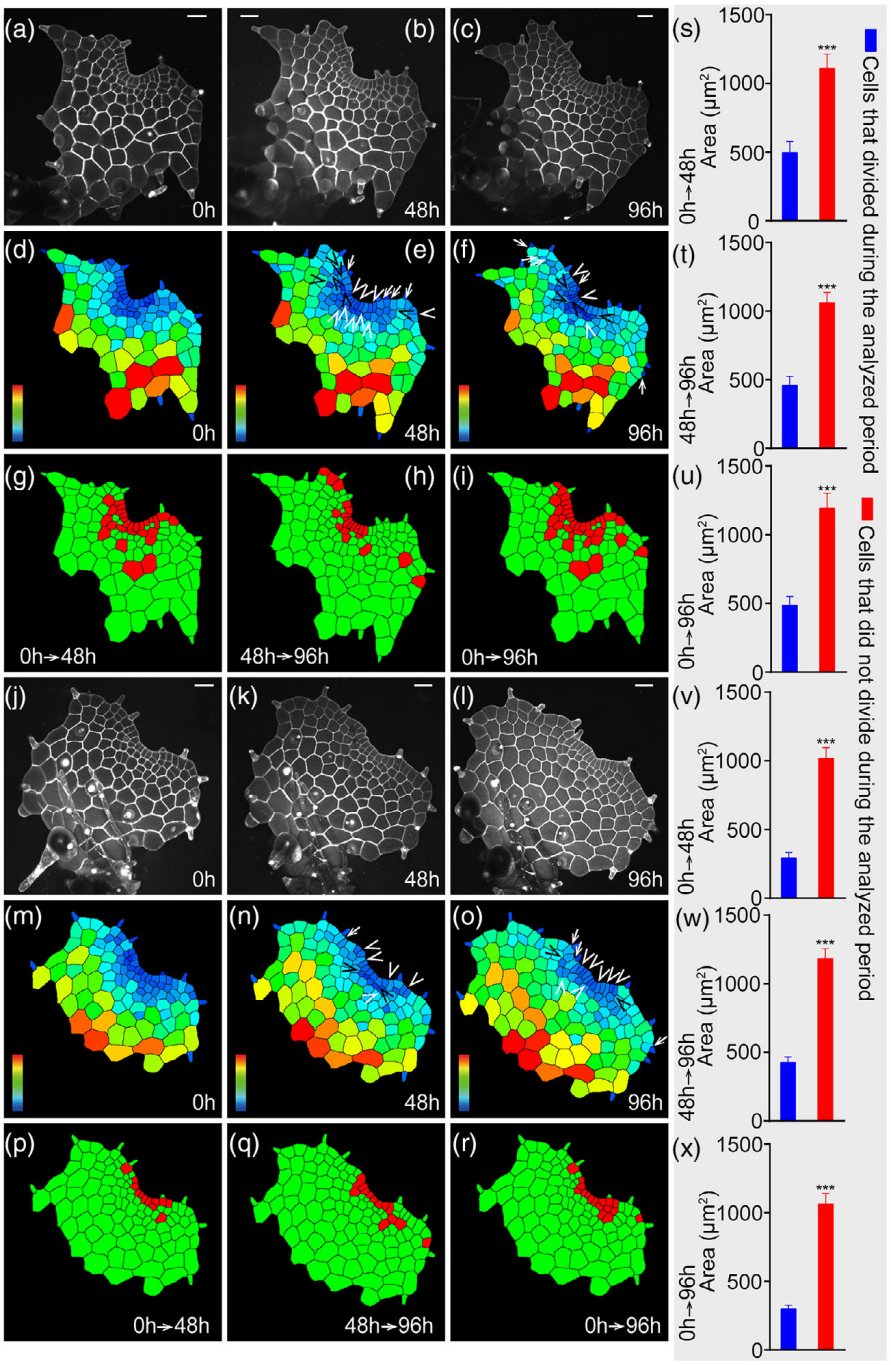
The patterns of cell division during apical notch formation in gametophytes. (a–r) Two *Woodsia obtusa* gametophytes (a–i, j–r) were stained and live‐imaged through laser scanning confocal microscopy at 0 h (a, j), 48 h (b, k), and 96 h (c, l). (d, e, f, m, n, o) Computational segmentation and cell size quantification of confocal images in (a, b, c, j, k, l), respectively. (g, h, p, q) Cell division in the gametophytes in (a, b, j, k) over 48 h. (i, r) Cell division in the gametophytes in (a, j) over 96 h. Cells that divided during the analyzed period are shown in red and the cells that did not divide during the analyzed period are shown in green. (a) The gametophyte at 30 days after inoculation (DAI). (j) The gametophyte at 31 DAI. At least three independent biological replicates showed similar patterns of cell division during notch formation. (s–x) Area quantification of the cells that divided and the cells that did not divide during the analyzed period, including the 0–48 h (s, v), 48–96 h (t, w), and 0–96 h (u, x) periods. Y‐axis: the averaged cell area. Bars: mean ± SE. In (s), *n* = 27 cells divided during the analyzed period and *n* = 73 cells did not divide during the analyzed period. In (t), *n* = 21 cells divided during the analyzed period and *n* = 103 cells did not divide during the analyzed period. In (u), *n* = 35 cells divided during the analyzed period and *n* = 65 cells did not divide during the analyzed period. In (v), *n* = 12 cells divided during the analyzed period and *n* = 87 cells did not divide during the analyzed period. In (w), *n* = 14 cells divided during the analyzed period and *n* = 97 cells did not divide during the analyzed period. In (x), *n* = 17 cells divided during the analyzed period and *n* = 82 cells did not divide during the analyzed period. (s–u) Cell area from the segmented images in (g–i), respectively. (v–x) Cell area from the segmented images in (p–r), respectively. Trichomes in (g–i, p–r) were not included in the area quantification in (s–x). (s–x) ***P < 0.001 (Student's two‐tailed *t*‐test). (a, b, c, j, k, l) Gray: propidium iodide (PI) stain; scale bar: 50 μm. Color bars in (d, e, f, m, n, o) indicate the quantified area of each segmented cell, with the scale ranging from blue (0) to red (≥3000 μm^2^). In (e, f, n, o), white ‘V’ indicates anticlinal division in multicellular meristems, black ‘V’ indicates periclinal division in multicellular meristems, and white arrows indicate cell division‐associated trichome development. The source data and statistical analysis for (s–x) are included in Tables S3–S8. [Colour figure can be viewed at wileyonlinelibrary.com]

**Figure 5:**
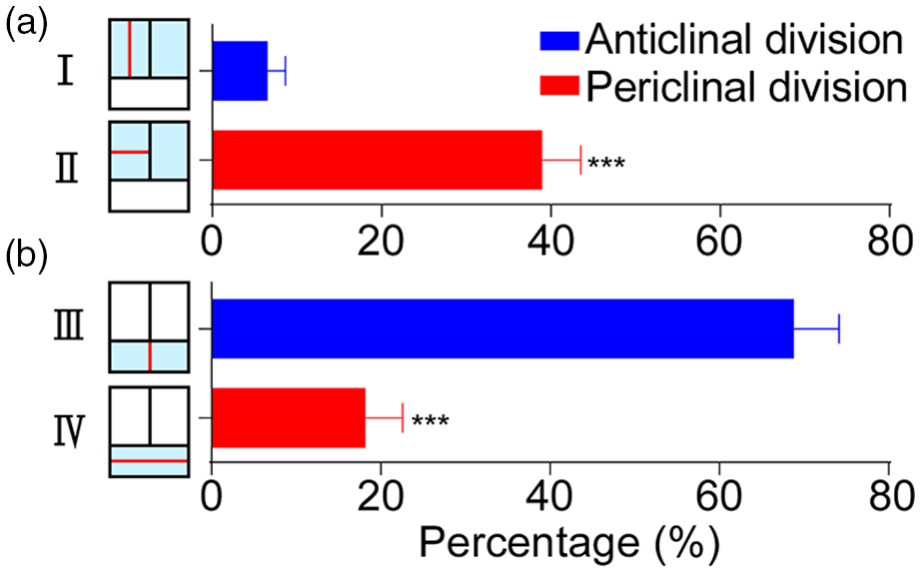
Quantification of different patterns of cell division in *Woodsia obtusa* gametophytes. (a, b) Y‐axis: four different patterns of cell division (I, II, III, IV) in the three‐celled packets, which are defined and summarized in Figure 2u. X‐axis: percentage of the defined packets showing the indicated type of cell division in the time‐lapse imaging experiments (*n* = 47 sets of time‐lapse experiments, which included 77 three‐celled packets from 34 independent samples in total). The equations for calculating the percentage of each division pattern are included and described in the ‘Materials and Methods’ section. One example of the defined three‐celled packets used for the analysis is included in Figure S13. Bars: mean ± SE. ***P < 0.001 (Student's two tailed *t*‐test). The source data and statistical analysis for this figure are included in Table S9. [Colour figure can be viewed at wileyonlinelibrary.com]

**Figure 6:**
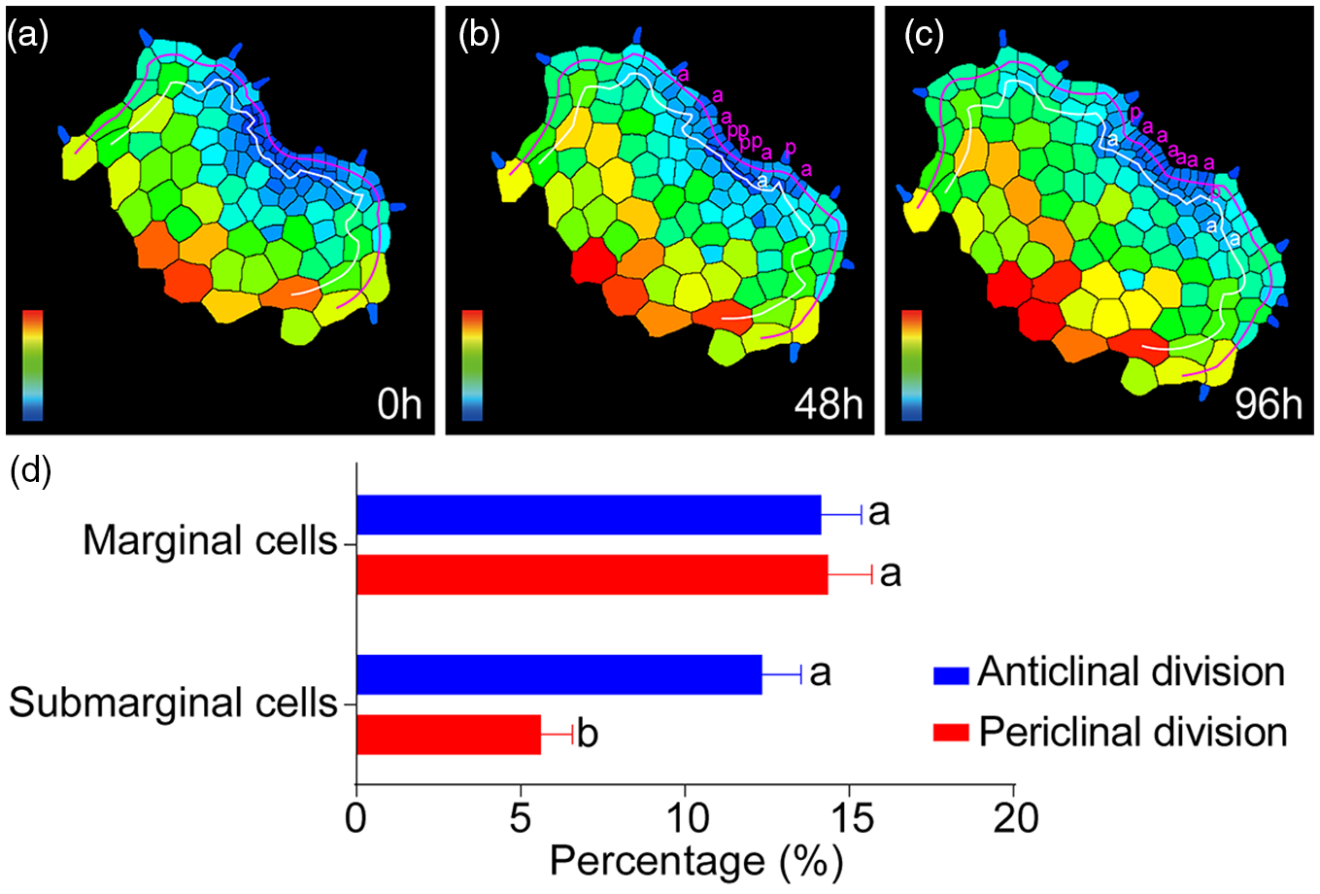
Quantification of cell division patterns in the marginal cells and the submarginal cells of *Woodsia obtusa* gametophytes. (a–c) Representative images (shown in Figure 4m–o) for calculating the percentages of cell division patterns. The purple line indicates the marginal cells and the white line indicates the submarginal cells which are included in the analysis. Each anticlinal (or periclinal) division is marked as a (or p) in (b, c). For cell division occurring between 0 and 48 h, the percentage of anticlinal (or periclinal) divisions in the marginal cells is calculated as number of a (or p) from the marginal cells at 48 h/number of marginal cells at 0 h, and the percentage of anticlinal (or periclinal) divisions in the submarginal cells is calculated as number of a (or p) from the submarginal cells at 48 h/number of submarginal cells at 0 h. For cell division occurring between 48 and 96 h, the percentage of anticlinal (or periclinal) divisions in the marginal cells is calculated as number of a (or p) from the marginal cells at 96 h/number of marginal cells at 48 h, and the percentage of anticlinal (or periclinal) divisions in the submarginal cells is calculated as number of a (or p) from the submarginal cells at 96 h/number of submarginal cells at 48 h. (d) Percentage of cells showing anticlinal (blue) or periclinal (red) division over 48 h. Bars: mean ± SE (*n* = 76 sets of time‐lapse experiments with 50 independent samples in total). Each set of time‐lapse experiments was quantified as the representative images shown in (a–c). Same letters indicate no significant statistical difference and different letters (a, b) indicate a statistically significant difference between two groups (two‐tailed *t*‐test, *P* < 0.001). The source data for Figure 6d are included in Table S10. [Colour figure can be viewed at wileyonlinelibrary.com]

**Figure 7:**
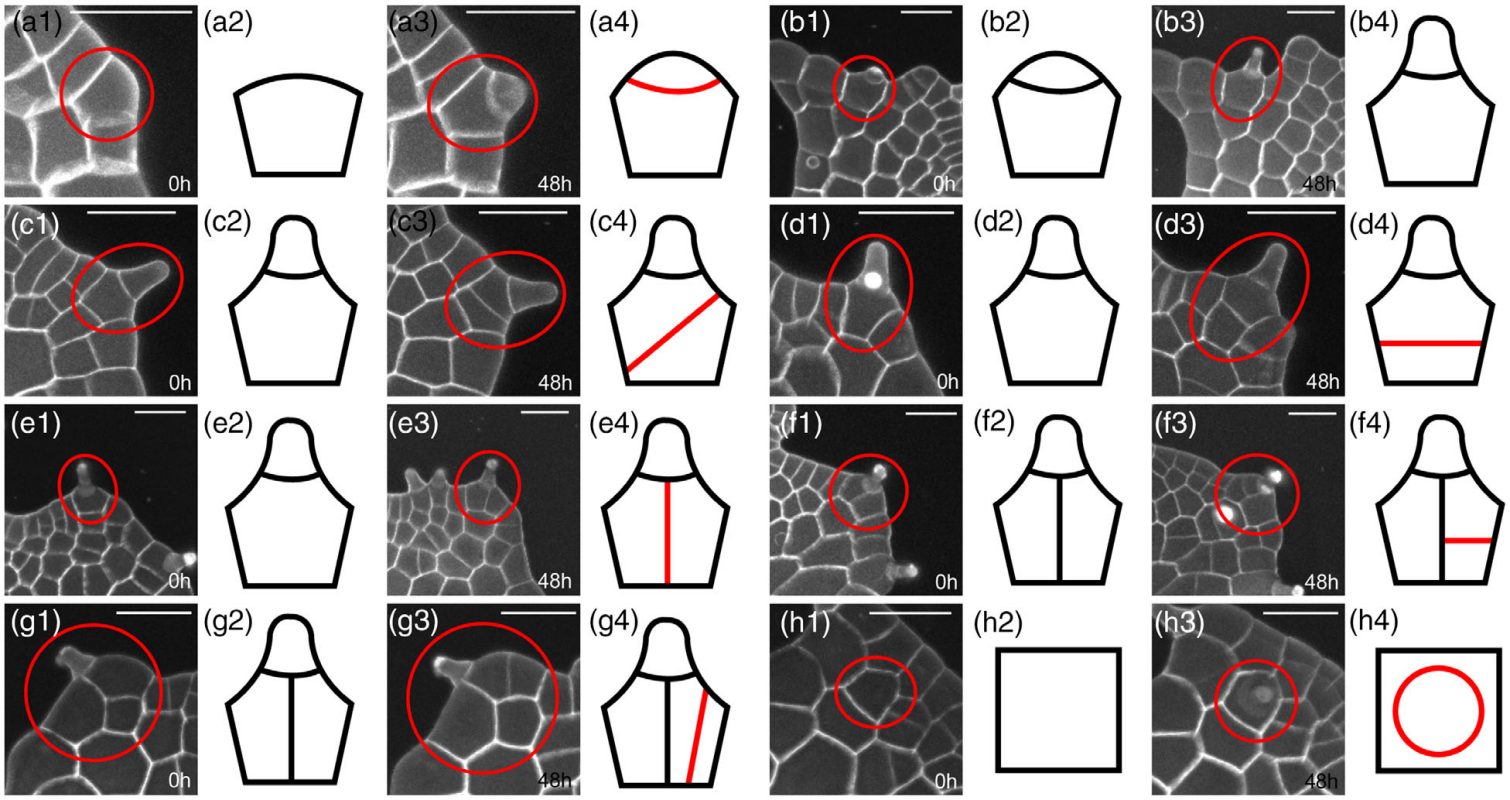
The patterns of cell division associated with trichome development in gametophytes. (a1–a4, b1–b4, c1–c4, d1–d4, e1–e4, f1–f4, g1–g4, h1–h4) Eight developing trichomes in *Woodsia obtusa* gametophytes were stained and live‐imaged through laser scanning confocal microscopy at 0 h (a1, b1, c1, d1, e1, f1, g1, h1) and 48 h (a3, b3, c3, d3, e3, f3, h3). (a1, c1, d1) The gametophytes at 23 days after inoculation (DAI). (g1, h1) The gametophytes at 28 DAI. (b1, e1, f1) The gametophytes at 30 DAI. Trichomes in (a1, a3, b1, b3, c1, c3, d1, d3, e1, e3, g1, g3, h1, h3) are from the gametophytes shown in Figures 2(a,c), 4(a,b), 1(p,r,f,h), 4(a,b), and 2(k,m,f,h), respectively. Trichomes in (b1–b4) and (e1–e4) are from the same gametophyte. (a2, a4, b2, b4, c2, c4, d2, d4, e2, e4, f2, f4, g2, g4, h2, h4) Diagrams and illustration of cell division patterns associated with trichome development, with newly formed cell wall shown in red. At least three independent biological replicates showing each pattern of cell division are illustrated. (a1, a3, b1, b3, c1, c3, d1, d3, e1, e3, f1, f3, g1, g3, h1, h3) Gray: propidium iodide (PI) stain; scale bar: 50 μm; red circles: developing trichomes and adjacent cells. [Colour figure can be viewed at wileyonlinelibrary.com]

We first focused on the morphology and division activity of an apical cell and its immediate progenies at an early stage of gametophyte development (Figure 1a–y; Figures S5–S7). At 23 DAI, a wedge‐shaped apical cell was located at the center of the marginal layer and it was morphologically distinguishable from all the other cells (Figure 1a,b,f,g,k,l,p,q; Figures S5a–d and S6a). The apical initial cell together with the adjacent cell formed a unique cell packet or cluster, which was also wedge‐shaped (indicated by the red circle in Figure 1p,q; Figures S5a,c and S6a and illustrated in Figure 1z; Figures S5e and S6b). Once specified, this wedge‐shaped cell packet self‐renewed through two rounds of cell division (Figure 1a–o,z; Figure S5a–e). Over a 48‐h interval, the wedge‐shaped apical cell from the packet formed a new cell wall obliquely through the center (Figure 1f–j,k–o,z; Figure S5). Such oblique division gave rise to two daughter cells with distinct shapes: a new wedge‐shaped apical cell and a flanking trapezoid‐shaped cell, forming a new two‐celled packet at the apex (Figure 1f–i,k–n; Figure S5d,e). In the meantime, the trapezoid‐shaped cell from the previously identified cell packet (Figure 1a,b,f,g; Figure S5a,c) underwent periclinal division, indicated by arrowheads (Figure 1d,i). Such periclinal division resulted in two short daughter cells with a similar trapezoid shape (Figure 1a–e,f–j,z; Figure S5), adjacent to the newly formed wedge‐shaped cell packet (Figure 1h,i; Figure S5d,e). These results from the time‐lapse imaging and image analysis showed that the apical initial in *W. obtusa* is able to renew itself through asymmetric, oblique division (Figure 1a–d,f–i,k–n; Figure S5), and the apical initial drives active cell proliferation (Figure 1e,j,o) at early stages of prothallus development.

### Patterns of cell division during the transition from an apical cell‐based meristem to a multicellular apical meristem

As shown in the snapshots, the wedge‐shaped apical cell in *W. obtusa* gametophytes was only maintained for a limited time during prothallus development, and it was eventually replaced by a multicellular apical meristem at the same region (Figure S1). We thus focused on the cell morphology and division during the termination of an apical initial (Figure 1p–y,z; Figures S6a–d and S7a–e). Different from the oblique division that yielded new wedge‐shaped cells (Figure 1f–i,k–n), in a wedge‐shaped apical cell, a periclinal division resulted in two short rectangle‐ or trapezoid‐shaped daughter cells (Figure 1p–y,z; Figures S6 and S7) and led to loss of the morphological signature of apical cells in their daughter cells, indicating the termination of the apical initial. In the meantime, the trapezoid‐shaped cell adjacent to the wedge‐shaped apical cell also underwent periclinal division, resulting in two short rectangle‐ or trapezoid‐shaped daughter cells (Figure 1p–s,z; Figure S6).

More importantly, over a 48‐h interval, we captured the patterns of cell division and growth that were specifically associated with the transition from an apical initial to a multicellular apical meristem (Figures 1u–y and 2a–t; Figure S7). Anticlinal division in the progenies of the apical cell (Figures 1u–x and 2a–j; Figure S7) led to multiple adjacent rectangular cells in the marginal layer (Figure 2a–e), consisting of a multicellular apical meristem with the morphology comparable to that of the previously characterized multicellular marginal meristems in *C*. *richardii* and *P. vittata* (Banks, 1999; Banks et al., 1993; Bartz & Gola, 2018; Conway & Di Stilio, 2020; Wu et al., 2021). Cell division was highly active (indicated by red color in Figure 2e,j,o,t) in the multicellular apical meristems compared to the cells outside of apical meristems, suggesting the multicellular meristem replaced the role of the apical initial (Figure 1e,j,o,t) to sustain the active cell proliferation at the apices of gametophytes.

At the apical region of the first two layers, we found the rectangular packets originating from three cells served as the building block and functional unit during the establishment of multicellular apical meristems in *W. obtusa* gametophytes (Figure 2a–d,f–i,k–n,p–s; see cartoon in Figure 2u; Figure S8). Each conserved pack consisted of two adjacent rectangular cells at the top and one short rectangular cell at the bottom (Figure 2u; Figure S8a1–a4). Although multicellular apical meristems in *W. obtusa* gametophytes were involved with variable patterns of cell proliferation, all of them can be classified into four basic types (I–IV) of cell division within each packet (Figure 2u; Figure S8b1–b4,c1–c4,d1–d4,e1–e4). Specifically, Type I showed an anticlinal cell division in the upper rectangular cell, which resulted in two adjacent elongated rectangular cells in the marginal layer (Figure 2u; Figure S8b1–b4). Type II showed a periclinal cell division in the upper rectangular cell within the packet, giving rise to one short rectangular cell outside and the other rectangular cell inside (Figure 2u; Figure S8c1–c4). Type III showed an anticlinal cell division at the lower rectangular cell, which led to two equally divided small rectangular cells at the bottom of the packet (Figure 2u; Figure S8d1–d4). In addition, Type IV represented a periclinal division at the lower rectangular cell, forming two even shorter rectangular cells at the bottom (Figure 2u; Figure S8e1–e4).

### Patterns of cell division during proliferation of multicellular apical meristems and formation of the apical notch

To further determine cell division dynamics during proliferation of multicellular meristems, we live‐imaged *W. obtusa* gametophytes at three time points over a 96‐h interval (Figures 3 and 4). At the late developmental stage (30 DAI, for example) when the multicellular apical meristem was first established, a cluster of elongated and rectangular cells, with the small sizes quantitatively indicated by the various shades of blue color, were located at the apical region of the first two layers (Figures 3d–f,m–o and 4d–f,m–o). These blue‐colored cells consisted of or largely overlapped with a multicellular apical meristem that eventually formed an apical notch.

During proliferation of multicellular apical meristems, the renewal and disappearance of three‐celled packets were dynamic, directly associated with different patterns of anticlinal and periclinal division (Figures 2u and 3a–r; Figures S8–S10). For example, within each rectangular cell packet, Type I resulted in two equally divided tall rectangular cells at the top and Type III resulted in two small rectangular cells at the bottom, and a combination of Type I and Type III resulted in a group of five cells (Figure S8f1–f4). In addition, Type II in the two adjacent cells resulted in four equally divided short rectangular cells at the top and Type III resulted in two equally divided small rectangular cells at the bottom, and together they resulted in a cluster of six cells (Figure 3j–o; Figure S8g1–g4). In other words, a combination of anticlinal and periclinal divisions (Types I–IV) led to the disappearance of three‐celled packets. In contrast, the conserved reverse ‘T’ pattern of cell division in one upper rectangular cell sustained the self‐renewal of the conserved three‐celled packets (Figure 3a–f). Specifically, this pattern consisted of a periclinal cell division followed by an anticlinal cell division (described in Imaichi, 2013 and Wu et al., 2021), resulting in three daughter cells from one rectangular cell. To comprehensively determine cellular dynamics of the three‐celled packets during meristem development, we further quantified the percentages of each of four types of division occurring in each packet (Figure 5a,b; Figure S13). Within the three‐celled packets, the upper rectangular cells had more periclinal cell division (Type II) than anticlinal cell division (Type I) (Figure 5a; Table S9). In contrast, the lower rectangular cells had more anticlinal division (Type III) than periclinal division (Type IV) (Figure 5b; Table S9).

Interestingly, during proliferation of multicellular apical meristems, wedge‐shaped cells were occasionally formed again at the apical region of the marginal layer. However, this morphology was not maintained after one or two rounds of cell division (Figure 3a–f; Figures S11 and S12). For example, one wedge‐shaped cell and the flanking trapezoid‐shaped cell formed a two‐celled group also in a wedge shape (Figure S11a). Over 96 h, cell division occurred in both cells in the periclinal orientation, resulting in short rectangle‐ or trapezoid‐shaped daughter cells (Figure S11), which was similar to the pattern involved in termination of apical initials (Figure 1p–t,z; Figure S6). In addition, the two‐celled group also formed four rectangular cells through cell division and expansion, eventually losing the wedge‐shaped cell (Figure 3a–f; Figure S12). These results demonstrate that once established, multicellular apical meristems maintained themselves through the renewal of three‐celled rectangular packets (Figure 2u; Figure S8), whereas the transiently formed wedge‐shaped cells quickly disappeared during gametophyte development (Figure 3a–f; Figures S11 and S12).

### The correlation between cell size and cell division activity in fern gametophytes

At late developmental stages (30–31 DAI), we found that cell division mainly occurred in small cells within the multicellular apical meristems (Figure 4d–i,m–r). To quantitively determine the correlation between cell size and cell division in fern gametophytes, we analyzed and compared the averaged sizes of the cells that divided and the cells that did not divide from two gametophytes after 48 or 96 h of growth (Figure 4s–x; Tables S3–S8). Our results showed that the size of the cells that divided was significantly smaller (*P* < 0.001) than that of the cells that did not divide during the analyzed time period (Figure 4s–x; Tables S3–S8). Since the small cell size was a feature of multicellular meristem proliferation in fern gametophytes (Figure 4d–f,m–o), these results link small cell size to high division activity of the meristem cells, suggesting an essential role of multicellular apical meristems in driving cell proliferation of *W. obtusa* gametophytes.

### Cell division in marginal cells and submarginal cells of fern gametophytes

To determine if the layer‐specific cell division occurs in the multicellular apical meristems of fern gametophytes, we first defined the cells from the marginal layer and from the submarginal layer in the live‐imaged *W. obtusa* gametophytes (Figure 6a–c, see ‘Materials and Methods’ section for details). Then, we quantified the percentages of anticlinal and periclinal divisions in the cells from the marginal layer or from the submarginal layer (Figure 6d; Figure S14, Tables S10 and S11). The quantitative results demonstrated that in *W. obtusa* gametophytes, the percentage of periclinal division in marginal cells was significantly higher than that in submarginal cells (Figure 6d, Table S10). In contrast, there was no significant difference between the percentage of anticlinal division in marginal cells and that in submarginal cells (Figure 6d, Table S10). Within the marginal layer, the percentages of anticlinal and periclinal division were also statistically comparable (Figure 6d; Figure S14a, Tables S10 and S11). In contrast, within the submarginal layer, the percentage of anticlinal division is significantly higher than that of periclinal division (Figure 6d; Figure S14b, Tables S10 and S11). All these quantifications demonstrate a unique division pattern in *W. obtusa* gametophytes, different from that in the outermost layers of shoot meristems in flowering plants (Meyerowitz, 1997).

### Cell growth and division during trichome development in gametophytes

Through time‐lapse imaging and image analysis, we also determined the patterns of cell proliferation that were directly associated with trichome initiation and proliferation in *W. obtusa* gametophytes. In the marginal layer, a rectangular cell underwent asymmetric division, leading to one small triangular daughter cell outside and one larger cell inside (Figure 7a1–a4). The triangular cell continued to expand outside (Figures 1a–d,f–i,k–n, 2a–d,k–n, 3j–o, 4a–f,j–o, and 7a1–a4; Figure S4a–d,f–i) and eventually formed a ‘pyramid‐shaped’ trichome at the surface of gametophytes (Figures 1f–i, 2k–n,p–s, 3a–f, 4a–f,j–o, and 7b1–b4). In addition, trichomes also initiated from inner cells of *W. obtusa* prothalli. Shown from the confocal Z‐projection view, daughter cells protruded out of the inner cells and developed into cone‐shaped trichomes, vertical to the mother cell layer (Figures 1a–d, 2f–i,k–n, and 7h1–h4; Figure S4a–d). The rectangular cells that were adjacent to the trichome cell continued to proliferate through different combinations of cell division. For example, oblique division occurred in the rectangular cells, resulting in two triangular daughter cells (Figures 1f–i,p–s,u–x and 7c1–c4). Alternatively, the periclinal or anticlinal division occurred in the rectangular cells that were adjacent to a developing trichome (Figures 1f–i, 2k–n,p–s, 4a–f,j–o, and 7d1–d4,e1–e4; Figure S4a–d). Following that, the derivatives were further divided by new wall, and the orientation of division was either perpendicular or parallel to the previous vertical wall (Figures 2a–d,k–n, 4a–f, and 7f1–f4,g1–g4; Figure S4f–i). Through this combination of cell division, trichomes continuously proliferated and did so without disturbing division and expansion of their adjacent cells in the marginal layer of gametophytes.

## DISCUSSION

### Apical initials: self‐renewal, termination, and transition to multicellular meristems

It has been proposed that a wedge‐shaped apical cell acts as the single initial to drive fern gametophyte development (Nayar & Kaur, 1971; Raghavan, 1989). Interestingly, the maintenance and activity of apical initials in gametophytes across fern taxa is highly diversified. For example, in the gametophytes of *C. richardii* (Pteridaceae) and *Anemia phyllitidis* (Anemiaceae), an apical cell is only transiently present, and its division activity was hardly observed (Banks, 1999; Bartz & Gola, 2018; Conway & Di Stilio, 2020; Takahashi et al., 2012). In contrast, through the quantification of cell division activity (Figure 1a–o), we found that the wedge‐shaped apical initial together with its immediate progenies serves as an active proliferation site during the early developmental stages in *W. obtusa* gametophytes. In addition, the pattern of oblique cell division seems to be highly conserved in renewing apical cells, because it has been repeatedly identified in the gametophytes of *W. obtusa* (Woodsiaceae) (Figure 1), *L. japonicum* (Lygodiaceae) (Takahashi et al., 2015), *P. vittata* (Pteridaceae) (Wu et al., 2021), and *Colysis decurrens* (Polypodiaceae) (Takahashi et al., 2009). Considering the distant relationship of these species in phylogeny (PPG, I., 2016), it is likely that functions of apical initials in gametophytes independently evolved among different orders of ferns. During gametophyte development, the apical initial in *W. obtusa* also loses its morphological signature and terminates itself through periclinal division (Figures 1 and 2). Eventually, an apical initial is directly replaced by a multicellular meristem at the apical region of *W. obtusa* gametophytes (Figures 1 and 2). Such transition between these two types of apical meristems in gametophytes is similar to that characterized in *L. japonicum* (Takahashi et al., 2015).

### Multicellular meristems: cell division patterns and activities

Multicellular meristems, including the multicellular apical meristem and the multicellular marginal meristem, seem to play a conserved role in sustaining gametophyte development in ferns (Banks, 1999; Banks et al., 1993; Bartz & Gola, 2018; Conway & Di Stilio, 2020; Imaichi, 2013; Nayar & Kaur, 1971; Takahashi et al., 2012, 2015; Wu et al., 2021). In *W. obtusa* gametophytes, the multicellular apical meristem drives apical notch formation through four types of anticlinal and periclinal cell divisions within the conserved three‐celled packets at late developmental stages (30–31 DAI, for example) (Figures 2u and 3, 4, 5, 6; Figure S8, Table S9). The conserved three‐celled packets renew themselves through the reverse ‘T’ pattern of cell division (Figure 3a–f), which is also highly conserved during multicellular meristem development in the gametophytes of several fern species examined (Bartz & Gola, 2018; Conway & Di Stilio, 2020; Takahashi et al., 2015; Wu et al., 2021). More importantly, studies combining the computational segmentation and area quantification show that the small cell size (indicated by blue color) is the conserved feature of multicellular meristems in the majority of gametophytes of *W. obtusa* (Figures 3d–f,m–o and 4d–f,m–o,s–x) and *P. vittata* (Wu et al., 2021); the active cell division is restricted to the multicellular meristem domain at late developmental stages (Figure 4g–i,p–r), further demonstrating the conserved role of multicellular meristems in driving cell proliferation during gametophyte development. Future work to identify the regulators controlling the cell division pattern and activity will provide more insights into multicellular meristem development in fern gametophytes.

### Meristems in fern gametophytes and shoot meristems in angiosperm sporophytes

Through the quantification of averaged sizes for the cells that divided or did not divide over 48 h, we found that small cells in fern gametophytes were more actively dividing in *W. obtusa* gametophytes (Figure 4s–x, Tables S3–S8). These results uncover the direct linkages among the cell location, cell size, and cell division activity during fern gametophyte development. These findings in ferns are different from cell proliferation in SAMs of flowering plants, where the cell division activity is not directly related to the cell size (Shapiro et al., 2015; Willis et al., 2016). Furthermore, our results demonstrate that the layer‐specific division pattern in meristems of fern gametophytes is distinct from that in shoot meristems of angiosperm sporophytes. In angiosperms (Arabidopsis, for example), the outermost layer (L1) of SAMs only undergoes anticlinal division, whereas the inner cell layers (corpus) of SAMs exhibit both anticlinal and periclinal division (Meyerowitz, 1997). In contrast, cells in the marginal layer of *W. obtusa* gametophytes divide in both anticlinal and periclinal orientations, and the percentages of marginal cells undergoing anticlinal or periclinal division were statistically similar (Figure 6d; Figure S14, Tables S10 and S11). In addition, this work (Figure 6d; Table S10) suggests a new proliferation model in *W. obtusa* gametophytes: both marginal cells and submarginal cells play comparable roles in increasing cell numbers in each layer (through anticlinal division); however, marginal cells likely make a larger contribution than submarginal cells to the increase in cell layers through periclinal division. It will be noteworthy to further test this model through quantitative studies in more fern species.

### New system for studying trichome development in gametophytes

Trichome formation in gametophytes is a conserved feature in many fern families, especially in the order Polypodiales (Nayar & Kaur, 1971). However, compared to the well‐characterized trichome initiation and differentiation in sporophytes of flowering plants (Hülskamp et al., 1994), the mechanism underlying trichome development in fern gametophytes was almost completely unknown. *Woodsia obtusa* gametophytes initiate trichomes at an early stage with only six or seven cells (Figure S2), and they continuously initiate and maintain multiple trichomes, likely independent of apical initials and multicellular apical meristems (Figures 1, 2, 3, 4 and 7; Figures S1, S2, and S4). The time‐lapse imaging analysis further revealed that the unique asymmetric division drives the initiation of trichomes from either marginal cells or inner cells in *W. obtusa* gametophytes (Figures 1, 2, 3, 4 and 7; Figures S2 and S4). Taken together, *W. obtusa* has the potential to serve as a new working model, not only for exploring fundamental cell behaviors during trichome development, but also for dissecting the communications between the differentiated (trichome) and undifferentiated (meristem) cells in fern gametophytes.

### Summary and perspectives

Using *W. obtusa* as a research system, we quantitively determined cell growth dynamics in two different types of apical meristems – the apical cell‐based meristem and the multicellular apical meristem – in fern gametophytes. Through comparison with previous findings in other fern species (Banks, 1999; Banks et al., 1993; Bartz & Gola, 2018; Conway & Di Stilio, 2020; Takahashi et al., 2009, 2012, 2015; Wu et al., 2021), our results suggest both conserved and unique patterns of cell division that drive meristem development in gametophytes. Considering the diversity of ferns and fern allies (Christenhusz & Byng, 2016; PPG, I., 2016; Sessa, 2018), the quantitative imaging platform that we have established will be valuable to further explore meristem evolution across different fern taxa and to comprehensively reveal cell behaviors in fern gametophytes in the future.

## MATERIALS AND METHODS

### Plant materials and growth condition

*Woodsia obtusa* spores (number 1987) were obtained from the American Fern Society. The spores of *W. obtusa* were sterilized with solution containing 2% bleach and 0.5% Tween 20 for 5 min and rinsed with sterile water six times. Spores were spread on fern medium (FM) with 0.5× MS salts (Sigma), pH 6.0, and 0.7% agar (Sigma). The gametophytes of *W. obtusa* were grown in a growth chamber (Percival) at 28°C with continuous light and 80% humidity. In the current growth condition and within the time frame examined (0–31 DAI), no gametangia or sexual dimorphism was observed in the *W. obtusa* gametophytes in this study. Spores show highly synchronous germination. Despite minor variations in prothallus size and total cell number, individual *W. obtusa* gametophytes with comparable morphology at the same or similar developmental stage showed comparable patterns of cell division and growth (Figures 1, 2, 3, 4, 5, 6; Figure S4).

### Confocal live imaging and image analysis

The gametophytes of *W. obtusa* were live‐imaged using a Zeiss LSM 880 upright confocal microscope. To determine the developmental dynamics of the *W. obtusa* gametophytes, snapshot images were taken from different gametophytes from 8 to 31 DAI. To determine the patterns of cell growth and division during the maintenance and termination of apical initials, the initiation and proliferation of multicellular apical meristems, and the initiation and proliferation of trichomes, non‐invasive time‐lapse confocal imaging was performed on gametophytes grown on fern medium (FM) plates at the indicated time points, following the procedure described in Wu et al., 2021 with minor modifications for this species. Specifically, one or two drops of freshly prepared propidium iodide (PI) solution (1 mg ml^−1^ in ddH_2_O) were directly applied to *W. obtusa* gametophytes grown on FM plates. After staining for 60 sec, the PI solution was quickly removed, and the samples were rinsed with excess amounts of sterilized water (ddH_2_O) at least two or three times. Then the stained gametophytes were live‐imaged directly on the FM plates. After imaging, the gametophytes were gently moved to new FM plates by pipette tips. The plates were moved back into the Percival growth chamber located next to the confocal microscope and cultured in the same conditions until the next time point. The confocal imaging settings were described previously (Geng & Zhou, 2019; Wu et al., 2021). Specifically, PI was excited by a 514‐nm laser and emission was detected from 571 to 651 nm. The gain was set within the range of 600–800 and the digital gain was 1. Other parameters in the Zeiss ZEN Black software were set as follows: scan mode (frame), scan area (512 × 512), averaging number (two), averaging method (mean), bit depth (16 bit), and scanning interval (1 μm). In the time‐lapse experiments, nine independent biological replicates were imaged at 0 and 24 h, 15 samples were imaged at 0 and 48 h, five samples were imaged at 0, 24, and 72 h, and 21 samples were imaged at 0, 48, and 96 h. The maximum‐intensity Z‐projection view of confocal images was generated using Fiji/ImageJ software (Schindelin et al., 2012). Gametophyte growth was quantified to determine the potential effect of PI staining on *W. obtusa* gametophyte development (Figure S15). At 25 DAI, 12 independent *W. obtusa* gametophytes were stained with PI solution as described above and 12 independent gametophytes were stained with water following the same procedure as the mock stain. After PI or mock staining, the solution was removed, and the gametophytes were rinsed with excess amounts of water three times and moved to new FM plates. All stained gametophytes on the FM plates were imaged through a stereoscope as 0 h, and they were moved back into the growth chamber and grown under identical conditions. At 48 h after staining, all gametophytes were imaged again through the stereoscope, and the area of each gametophyte (excluding trichomes) at 0 h and 48 h was measured through ImageJ/Fiji. The growth rate of each individual gametophyte over 48 h was calculated as the gametophyte area (at 48 h)/the gametophyte area (at 0 h). The two‐tailed *t*‐test was performed to determine the statistical difference between the growth rates of the PI‐stained and mock‐stained samples. The result (Figure S15) demonstrated that the PI staining procedure used in this study did not lead to a statistically significant inhibitory effect on gametophyte development during the analyzed time period in *W. obtusa*.

Because each *W. obtusa* gametophyte develops a flat sheet of cells, 2D image segmentation of the confocal images was performed, using the 2D watershed method described previously (Vincent & Soille, 1991). The area of each segmented cell was quantified using MATLAB software using the established procedure reported in Wu et al., 2021. The code is available upon request. Figure S3 showed the process of the segmentation and quantification of one representative set of time‐lapse images. The area of each segmented cell from an individual sample was quantitatively indicated by color with the scale specified in each figure legend. The quantified cell area from one representative sample (at 0 and 48 h) is shown in Tables S1 and S2. All the cells that divided or did not divide during the analyzed period were determined based on the segmented time‐lapse images and labeled using MATLAB. The dataset for quantifying the averaged cell area in the cells that divided and the cells that did not divide during the analyzed time periods is included in Tables S3–S8.

### Quantification of division patterns and statistical analysis

The three‐celled rectangular packet within a multicellular apical meristem was determined as the representative images shown in Figure S13(a–c), all showing the conserved organization with two upper rectangular cells and one lower rectangular cell at the starting time point. In total, 77 three‐celled packets were identified from the live‐imaged samples and included in the analysis of cell division shown in Figure 5a,b.

The percentage of each type of division in three‐celled packets was calculated based on the following equations:

$$\text{Percentage of Type}\;I\;\text{division}=\text{Number of Type}\;I\;\text{division}/\text{Cell number}\;\left(2\right)\times 100\%.$$

$$\text{Percentage of Type}\;\mathrm{II}\;\text{division}=\text{Number of Type}\;\mathrm{II}\;\text{division}/\text{Cell number}\;\left(2\right)\times 100\%.$$

Because Type I and Type II represented cell division in the upper two adjacent rectangular cells, in these two equations, the number of cell division events ranged from 0 to 2, and the cell number was 2.

$$\text{Percentage of Type}\;\mathrm{III}\;\text{division}=\text{Number of Type}\;\mathrm{III}\;\text{division}/\text{Cell number}\;\left(1\right)\times 100\%.$$

$$\text{Percentage of Type}\;\mathrm{IV}\;\text{division}=\text{Number of Type}\;\mathrm{IV}\;\text{division}/\text{Cell number}\;\left(1\right)\times 100\%.$$

Because Type III and Type IV represented cell division in the lower short rectangular cell, in these two equations, number of cell division events was 0 or 1, and the cell number was 1. When a three‐celled packet contained any cell undergoing more than one round of divisions within the imaging frame, it represented the complex combination of basic division types, and it was not included in the calculations shown in Figure 5.

Three cells (two upper cells and one lower cell) in each three‐celled packet were included and calculated for the four different types (Figure S8f1–f4,g1–g4). Therefore, the total percentages of Type I, Type II, Type III, and Type IV calculated were more than 100%. The datasets for calculating each type of division in 77 three‐celled packets is included in Table S9. The two‐tailed *t*‐test was performed; the *P*‐values are included in Table S9.

The marginal cells and submarginal cells of gametophytes were determined as shown in the representative images in Figure 6(a–c). To determine the percentage of cells with anticlinal division or periclinal division per 48 h (Figure 6d), the time‐lapse images from 0 to 48 h were included in the analysis (Table S10). The time‐lapse images from 0 to 24 h were also included, and the data were normalized to the 48 h time frame (Table S10). The images with three time points (0, 48, and 96 h) were analyzed every 48 h and were calculated as two independent sets of 48 h: one from 0 to 48 h and the other from 48 to 96 h. Submarginal cells were defined as one layer of cells that were in direct contact with marginal cells. For example, over the 0–48 h time frame, the cell added inwards after periclinal division of the marginal cell was defined as the new submarginal cell when calculating the division patterns within the following 48–96 h time frame. In addition, over the 0–48 h time frame, the cell added inwards after periclinal division of the submarginal cell was not included as a submarginal cell within the following 48–96 h time frame.

In total, 1331 cells from the marginal layer and 983 cells from the submarginal layer were included in the analysis shown in Figure 6d. The percentage of anticlinal or periclinal cell division every 48 h in all the marginal or submarginal cells was calculated based on the following equations:

Percentage of anticlinal cell division every48hin marginal cells=Number of anticlinal divisions in marginal cells/Number of marginal cellsbefore the imaging/Time frameh×48h×100%.

Percentage of periclinal cell division every48hin marginal cells=Number of periclinal divisions in marginal cells/Number of marginal cellsbefore the imaging/Time frameh×48h×100%.

Percentage of anticlinal cell division every48hin submarginal cells=Number of anticlinal divisions in submarginal cells/Number of submarginal cellsbefore the imaging/Time frameh×48h×100%.

Percentage of periclinal cell division every48hin submarginal cells=Number of periclinal divisions in submarginal cells/Number of submarginal cellsbefore the imaging/Time frameh×48h×100%.

Within these equations, the time frames for each biological replicate are 24 or 48, which are listed in Table S10. The dataset for calculating the percentage of each division in all marginal cells and submarginal cells is included in Table S10. The two‐tailed *t*‐test was performed to determine the statistical difference between the datasets of each two types of division; the *P*‐values are included in Table S10.

In addition, the percentages of anticlinal or periclinal cell division in the marginal cells that divided or in the submarginal cells that divided during the analyzed period were also calculated based on the following equations:

Percentage of anticlinal cell division in the marginal cellsthat divided during the analyzed period=Number of anticlinal divisions in the marginal cells/total number of cell division events in the marginalcells during the analyzed time period×100%.

Percentage of periclinal cell division in the marginal cellsthat divided during the analyzed period=Number of periclinal divisions in the marginal cells/total number of cell division events in the marginalcells during the analyzed time period×100%.

Percentage of anticlinal cell division in the submarginalcells that divided during the analyzed period=Number of anticlinal divisions in the submarginal cells/total number of cell division events in the submarginalcells during the analyzed time period×100%.

Percentage of periclinal cell division in the submarginalcells that divided during the analyzed period=Number of periclinal divisions in the submarginal cells/total number of cell division events in the submarginalcells during the analyzed time period×100%.

The datasets for calculating the percentages of anticlinal or periclinal division in the marginal cells that divided or in the submarginal cells that divided during the analyzed period are included in Table S11. The two‐tailed *t*‐test was performed to determine the statistical difference between the datasets of two types of division; the *P*‐values are included in Table S11. When one marginal or submarginal cell showed more than one cell division event during the analyzed time period, all cell division events (in anticlinal or periclinal orientations) within the analyzed time period were included in the calculations shown in Figure 6d, Figure S14, and Tables S10 and S11.

## AUTHOR CONTRIBUTIONS

YZ conceived the research; XW and XY performed the experiments; JAB and YZ supervised the experiments at Purdue; XW, XY, JAB, SZ, and YZ discussed the experimental results; AY, XW, and YZ performed computational analysis and quantification; XW and YZ wrote the manuscript; and JAB, AY, and SZ revised the manuscript. All authors approved the manuscript. The authors declare no conflict of interest.

## Supporting information


**Figure S1.** The morphology of *Woodsia obtusa* gametophytes.
**Figure S2.** Confocal imaging of *Woodsia obtusa* gametophytes at early developmental stages.
**Figure S3.** Computational segmentation and quantification of confocal time‐lapse images.
**Figure S4.** The patterns of cell divisions associated with trichome development in gametophytes.
**Figure S5.** Confocal imaging and illustration of cell division patterns during proliferation of apical initials.
**Figure S6.** Confocal imaging and illustration of cell division patterns during termination of apical initials.
**Figure S7.** Confocal imaging and illustration of cell division patterns after termination of apical initials.
**Figure S8.** Confocal imaging and illustration of cell division patterns during initiation and proliferation of multicellular apical meristems in gametophytes.
**Figure S9.** Confocal imaging and illustration of cell division patterns during proliferation of multicellular meristems.
**Figure S10.** Confocal imaging and illustration of cell division patterns during proliferation of multicellular meristems.
**Figure S11.** Confocal imaging and illustration of the cell division patterns that lead to disappearance of wedge‐shaped cells during proliferation of multicellular meristems.
**Figure S12.** Disappearance of wedge‐shaped cells during proliferation of multicellular meristems.
**Figure S13.** Representative images showing the defined three‐celled packets from one sample at different time points.
**Figure S14.** Quantification of cell division patterns in the (a) marginal and (b) submarginal cells that divided during the analyzed time period.
**Figure S15.** The growth rate of *Woodsia obtusa* gametophytes over 48 h after mock or PI staining.

**Table S1.** Area quantification of each segmented cell from the *Woodsia obtusa* gametophyte shown in Figure S3(a–c) and Figure 1(u).
**Table S2.** Area quantification of each segmented cell from the *Woodsia obtusa* gametophyte shown in Figure S3(d–f) and Figure 1(w).
**Table S3.** The source data for Figure 4(s).
**Table S4.** The source data for Figure 4(t).
**Table S5.** The source data for Figure 4(u).
**Table S6.** The source data for Figure 4(v).
**Table S7.** The source data for Figure 4(w).
**Table S8.** The source data for Figure 4(x).
**Table S9.** Summary of percentages of four types of division in 77 three‐celled packets during the proliferation of multicellular meristems in *Woodsia obtusa* gametophytes.
**Table S10.** Summary of cell division patterns in all the marginal cells and submarginal cells of *Woodsia obtusa* gametophytes.
**Table S11.** Summary of cell division patterns in the marginal cells and submarginal cells that divided during the analyzed period in *Woodsia obtusa* gametophytes.

## Data Availability

All the data were included in the manuscript and the supplementary files.
