# Supplementary material for: Cell growth dynamics in two types of apical meristems in fern gametophytes
Source: Plant J. 2022 May 12;111(1):149–63. doi: 10.1111/tpj.15784 (PMC9541313; doi:10.1111/tpj.15784)
Supplement: Supplementary file 1 — Figure S1. The morphology of Woodsia obtusa gametophytes. Figure S2. Confocal imaging of Woodsia obtusa gametophytes at early developmental stages. Figure S3. Computational segmentation and quantification of confocal time‐lapse images. Figure S4. The patterns of cell divisions associated with trichome development in gametophytes. Figure S5. Confocal imaging and illustration of cell division patterns during proliferation of apical initials. Figure S6. Confocal imaging and illustration of cell division patterns during termination of apical initials. Figure S7. Confocal imaging and illustration of cell division patterns after termination of apical initials. Figure S8. Confocal imaging and illustration of cell division patterns during initiation and proliferation of multicellular apical meristems in gametophytes. Figure S9. Confocal imaging and illustration of cell division patterns during proliferation of multicellular meristems. Figure S10. Confocal imaging and illustration of cell division patterns during proliferation of multicellular meristems. Figure S11. Confocal imaging and illustration of the cell division patterns that lead to disappearance of wedge‐shaped cells during proliferation of multicellular meristems. Figure S12. Disappearance of wedge‐shaped cells during proliferation of multicellular meristems. Figure S13. Representative images showing the defined three‐celled packets from one sample at different time points. Figure S14. Quantification of cell division patterns in the (a) marginal and (b) submarginal cells that divided during the analyzed time period. Figure S15. The growth rate of Woodsia obtusa gametophytes over 48 h after mock or PI staining. [file TPJ-111-149-s001.pdf]

Supplementary files

Figures S1-S15

Tables S1-S11

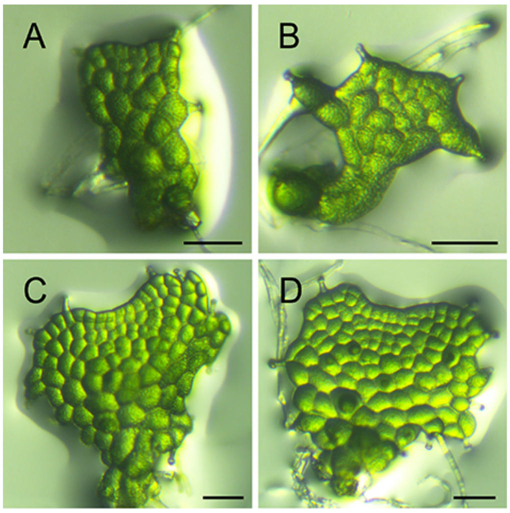

Figure S1. The morphology of *Woodsia obtusa* gametophytes. (A-D) Representative images of *Woodsia obtusa* gametophytes at 24 (A, B) and 38 (C, D) days after inoculation (DAI). Scale bar: 100 µm (A-D). At least three independent biological replicates show the morphology comparable to each representative snapshot included in the figure.

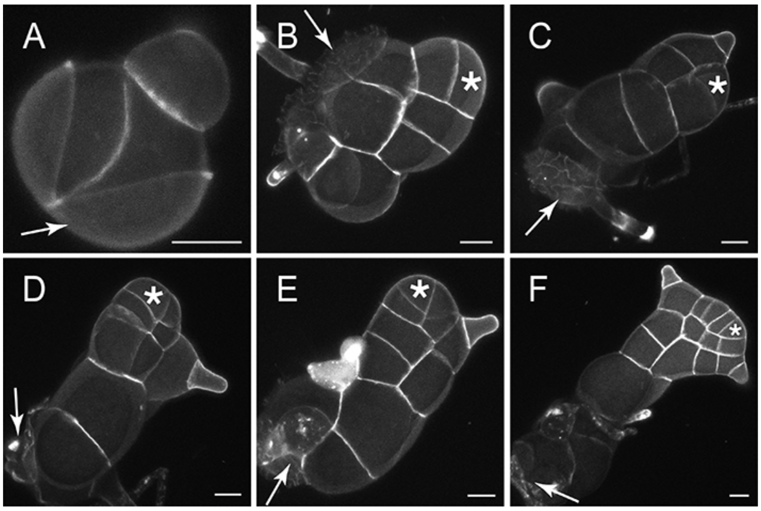

Figure S2. The confocal imaging of *Woodsia obtusa* gametophytes at early developmental stages. (A-F) six gametophytes were stained and imaged through the laser scanning confocal microscopy at 8 (A), 16 (B), 17 (C, D), 20 (E) and 22 (F) DAI. At least three independent biological replicates showed the growth pattern comparable to each representative snapshot included in the figure. (A-F) Grays: propidium iodide (PI) stain; Scale bar: 20 μm. Stars indicate the wedge-shaped apical initials and white arrows indicate the spore coat.

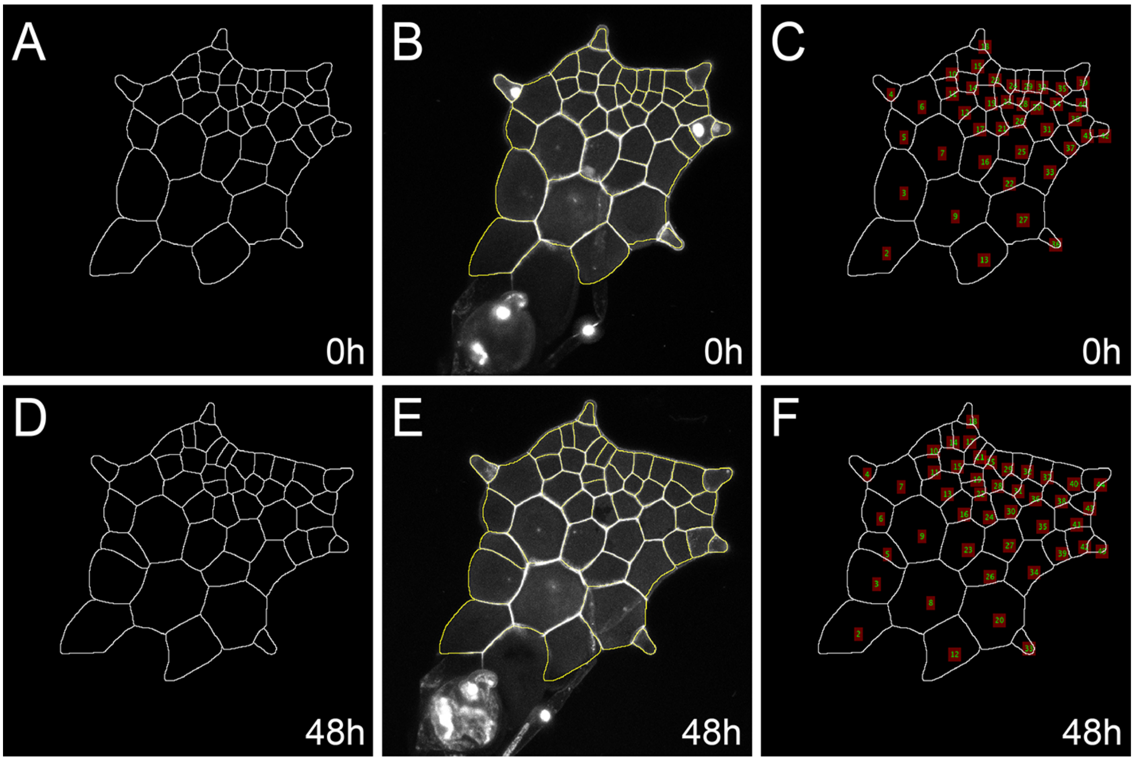

Figure S3. Computational segmentation and quantification of confocal time-lapse images. (A, D) the segmented images of one representative gametophyte at 0h (in Figure 1U) an 48h (in Figure 1W) are shown here as an example. (B, E) The overlay of the confocal image (gray) and the computational segmentation (yellow) of cell wall. (C, F) Each segmented cell is labelled with a unique ID in the gametophyte and the area of each segmented cell is automatically quantified. The quantitative results are included in Tables S1, S2. All time-lapse confocal images in Figures 1-4, S4 were analyzed and quantified using the same procedure.

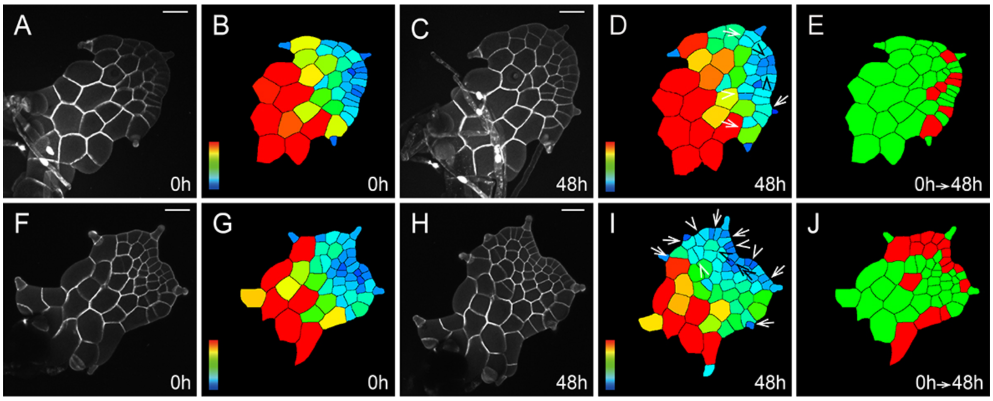

Figure S4. The patterns of cell divisions associated with trichome development in gametophytes. Two *W. obtusa* gametophytes (A-E, F-J) were stained and live imaged through the laser scanning confocal microscopy at 0 hour (A, F) and 48 hours (C, H). (B, D, G, I) show the computational segmentation and cell size quantification of confocal images in (A, C, F, H), respectively. (E, J) highlight cell division in the gametophytes (A, F), with the cells that divided during the analyzed time period (48 hours) in red and the cells that did not divide during the same time period in green. (F) shows the gametophyte at 25 DAI and (A) shows the gametophyte at 28 DAI. (A, C, F, H) Grays: propidium iodide (PI) stain; Scale bar: 50 μm. Color bars (in B, D, G, I) indicate quantified area of each segmented cell, with the scale from blue (0) to red (at or above 2000 μm^2). (B, D, G, I) White 'V' indicates the anticlinal division in multicellular apical meristems, black 'V' indicates the periclinal division in multicellular meristems and white arrows indicate cell division associated with trichome development.

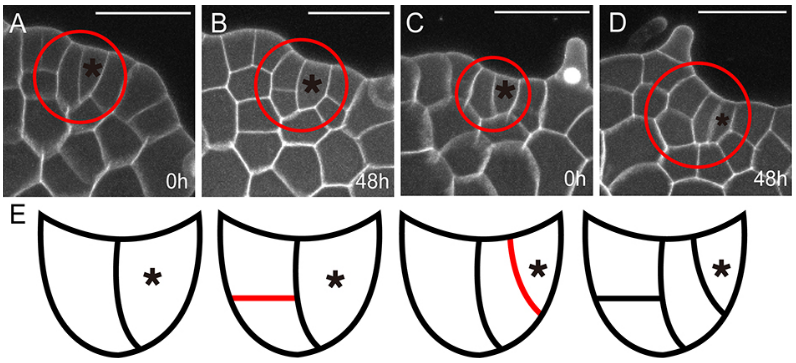

Figure S5. Confocal imaging and illustration of cell division patterns during proliferation of apical initials. The apical initials from two *W. obtusa* gametophytes (A-B, C-D) were stained and live imaged through the laser scanning confocal microscopy at 0 hour (A, C) and 48 hours (B, D). (A, C) show the gametophytes at 23 DAI. The apical initials shown in Figure S5A, B, C, D are from the gametophytes shown in Figure 1A, C, F, H. (E) Diagrams and illustration of cell division patterns during the proliferation of apical initials, with newly formed cell wall in red. At least three independent biological replicates showed similar patterns of cell divisions during the proliferation of apical initials. (A, B, C, D) Grays: propidium iodide (PI) stain; Scale bar: 50 µm. (A, B, C, D) Red circles highlight the cell packets containing the apical initial and its immediate progenies, and stars indicate the wedge-shaped apical initials.

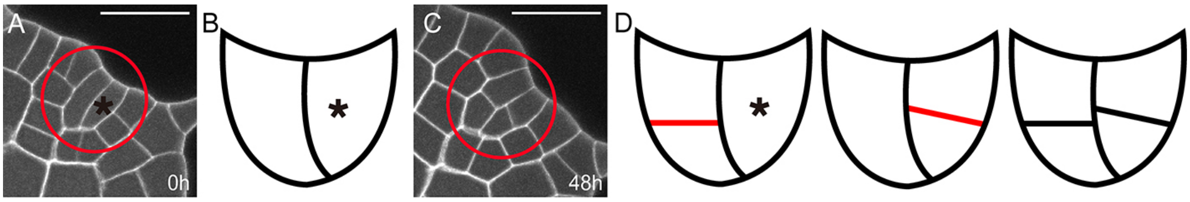

Figure S6. Confocal imaging and illustration of cell division patterns during termination of apical initials. One *W. obtusa* gametophyte (A, C) was stained and live imaged through the laser scanning confocal microscopy at 0 hour (A) and 48 hours (C). (A) shows the gametophytes at 23 DAI. The highlighted cell packets in Figure S6A, C are from the gametophyte shown in Figure 1P, R. (B, D) Diagrams and illustration of cell division patterns during termination of apical initials, with newly formed cell wall in red. At least three independent biological replicates showed similar patterns of cell divisions during termination of apical initials. (A, C) Grays: propidium iodide (PI) stain; Scale bar: 50 μm. (A, C) Red circles represent cell packets containing the apical initial and its immediate progenies, and (A, C, D) stars indicate the wedge-shaped apical initials.

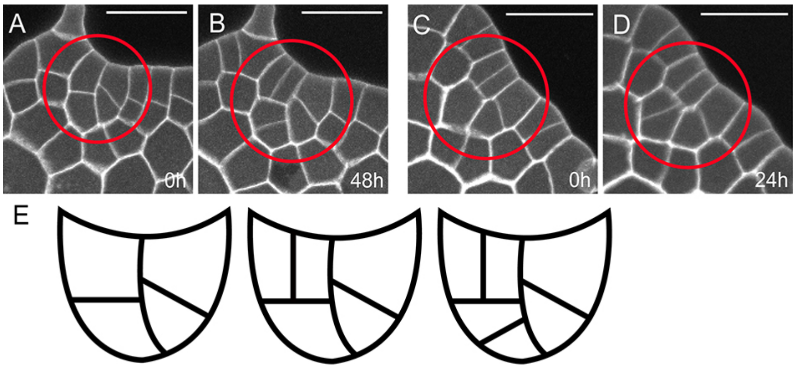

Figure S7. Confocal and illustration of cell division patterns after termination of apical initials. Two *Woodsia obtusa* gametophytes (A-B, C-D) were stained and live imaged through the laser scanning confocal microscopy at 0 hour (A, C), 24 hours (D) and 48 hours (B). (A) shows the gametophytes at 23 DAI and (C) shows the gametophytes at 24 DAI. The highlighted cell packets shown in Figure S7 A, B are from the gametophyte shown in Figure 1U, and 1W. (E) Diagrams and illustration of conserved cell packets after termination of apical initials. (A, B, C, D) Grays: propidium iodide (PI) stain; Scale bar: 50 µm. (A, B, C, D) Red circles represent the conserved cell packets.

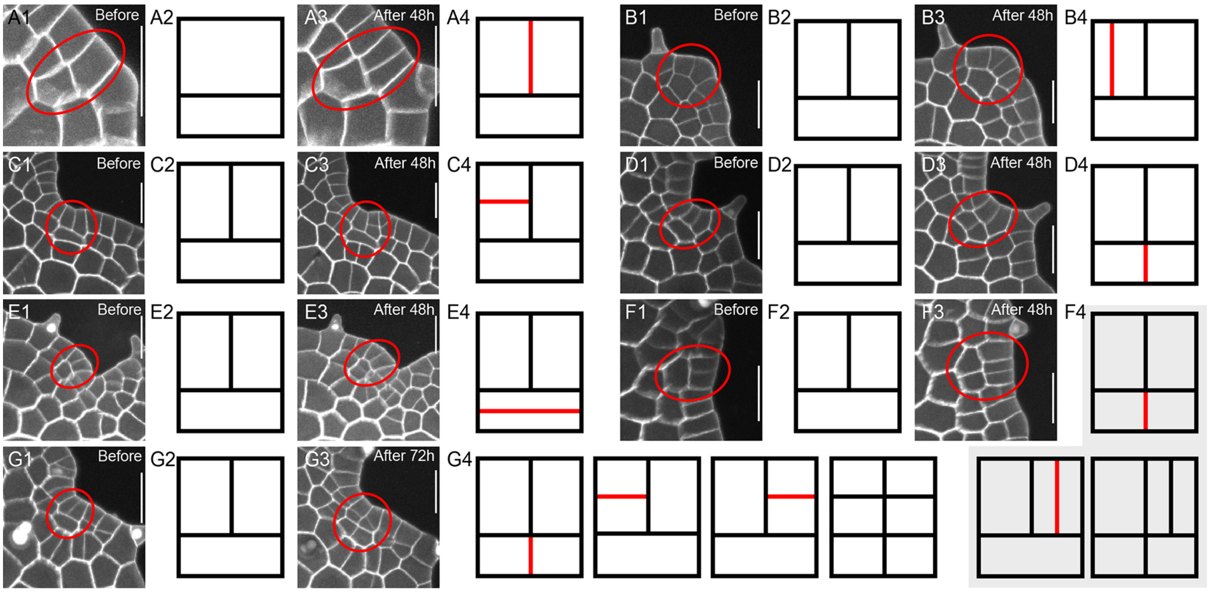

Figure S8. Confocal imaging and illustration of cell division patterns during initiation and proliferation of multicellular apical meristems in gametophytes. Seven *W. obtusa* gametophytes (A1-A4, B1-B4, C1-C4, D1-D4, E1-E4, F1-F4, G1-G4) were stained and live imaged through the laser scanning confocal microscopy before (A1, B1, C1, D1, E1, F1, G1) and after 48 hours (A3, B3, C3, D3, E3, F3) or after 72 hours (G3). (A1) shows the gametophytes at 23 DAI, (F1) shows the gametophytes at 28 DAI, (G1) shows the gametophytes at 29 DAI, (B1, D1) show the gametophytes at 30 DAI, and (C1, E1) show the gametophytes at 31 DAI. The highlighted cell packets shown in Figure S8 A1, A3, G1, G3 are from the gametophytes shown in Figures 2A, 2C, 3J, 3L. (A2, A4, B2, B4, C2, C4, D2, D4, E2, E4, F2, F4, G2, G4) Diagrams and illustration of cell division patterns during initiation and proliferation of multicellular apical meristems in gametophytes, with newly formed cell wall in red. At least three independent biological replicates showed each type of cell division patterns illustrated. (A1, A3, B1, B3, C1, C3, D1, D3, E1, E3, F1, F3, G1, G3) Grays: propidium iodide (PI) stain; Scale bar: 50 μm. (A1, A3, B1, B3, C1, C3, D1, D3, E1, E3, F1, F3, G1, G3) Red circles represent the conserved three-celled packets.

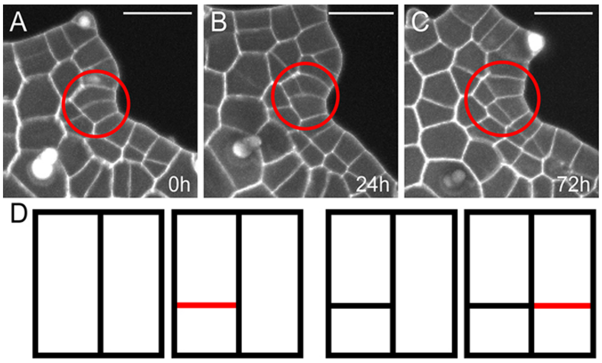

Figure S9. Confocal imaging and illustration of cell division patterns during proliferation of multicellular meristems. One *W. obtusa* gametophyte (A-C) was stained and live imaged through the laser scanning confocal microscopy at 0 hour (A), 24 hours (B) and 72 hours (C). (A) shows the gametophytes at 29 DAI. The highlighted cell packets shown in Figure S9A, B, C are from the gametophytes shown in Figure 3J, K, L. (D) Diagrams and illustration of cell division patterns during proliferation of multicellular meristems in gametophytes, with newly formed cell wall in red. At least three independent biological replicates showed each type of cell division patterns illustrated. (A1, A3, B1, B3, C1, C3, D1, D3, E1, E3, F1, F3, G1, G3) Grays: propidium iodide (PI) stain; Scale bar: 50 µm. (A, B, C) Red circles represents the conserved cell packets.

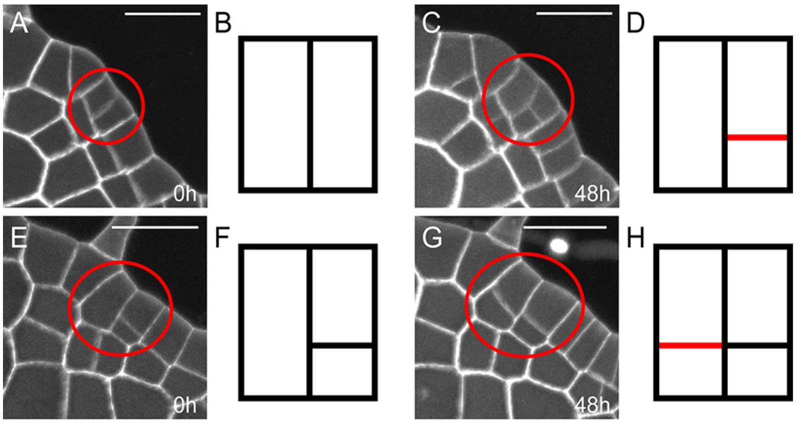

Figure S10. Confocal imaging and illustration of cell division patterns during proliferation of multicellular meristems. Two *Woodsia obtusa* gametophytes (A-D, E-H) were stained and live imaged through the laser scanning confocal microscopy at 0 hour (A, E) and 48 hours (C, G). (A) shows the gametophyte at 30 DAI, and (E) shows the gametophyte at 31 DAI. (B, D, F, H) Diagrams and illustration of cell division patterns during proliferation of multicellular meristems in gametophytes, with newly formed cell wall in red. At least three independent biological replicates showed each type of cell division patterns illustrated. (A, C, E, G) Grays: propidium iodide (PI) stain; Scale bar: 50 µm. (A, C, E, G) Red circles represents the conserved cell packets.

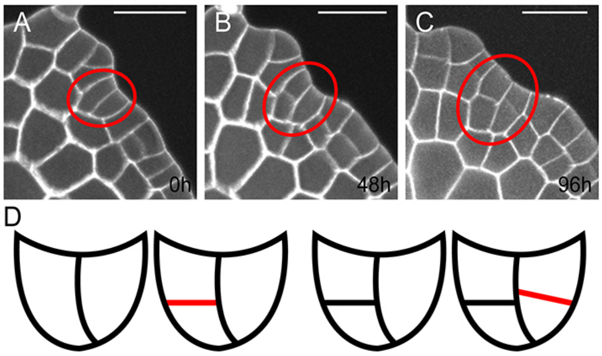

Figure S11. Confocal imaging and illustration the cell division patterns that lead to disappearance of wedge-shaped cells during proliferation of multicellular meristems. One *W. obtusa* gametophyte (A-C) was stained and live imaged through the laser scanning confocal microscopy at 0 hour (A), 48 hours (B) and 96 hours (C). (A) shows the gametophyte at 31 DAI. (D) Diagrams and illustration of cell division patterns during proliferation of multicellular meristems in gametophytes, with newly formed cell wall in red. At least three independent biological replicates showed similar patterns of cell division. (A, B, C) Grays: propidium iodide (PI) stain; Scale bar: 50 µm. (A, B, C) Red circles represent the conserved cell packets.

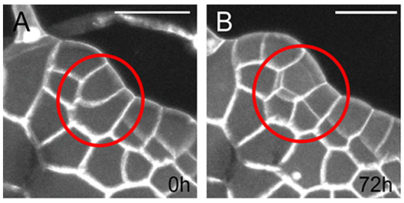

Figure S12. Disappearance of wedge-shaped cells during proliferation of multicellular meristems. One *W. obtusa* gametophyte (A, B) was stained and live imaged through the laser scanning confocal microscopy at 0 hour (A) and 72 hours (B). (A) shows the gametophyte at 29 DAI. The highlighted cell packets shown in Figure S12A, B are from the gametophytes shown in Figure 3A, C. (A, B) Grays: propidium iodide (PI) stain; Scale bar: 50 µm. (A, B) Red circles highlight the cell group contain a wedge-shaped cell.

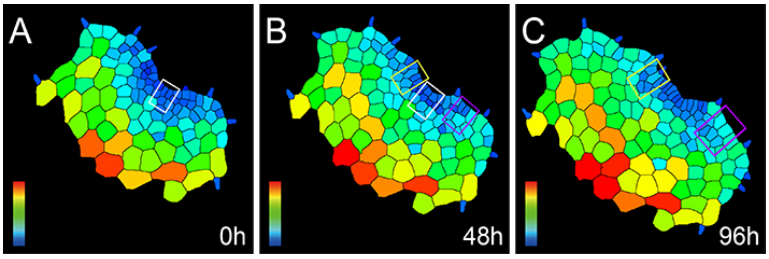

Figure S13. The representative images showed the defined three-celled packets from one sample at different time points. (A-C) Rectangular frames indicate the defined three-celled packets from the time-lapse images. Different colors of rectangular frames indicate different packets of three cells. The images of one representative gametophyte (in Figure 4M-O) were labelled here, and all the other samples and packets were determined similarly for the quantification shown in Figure 5.

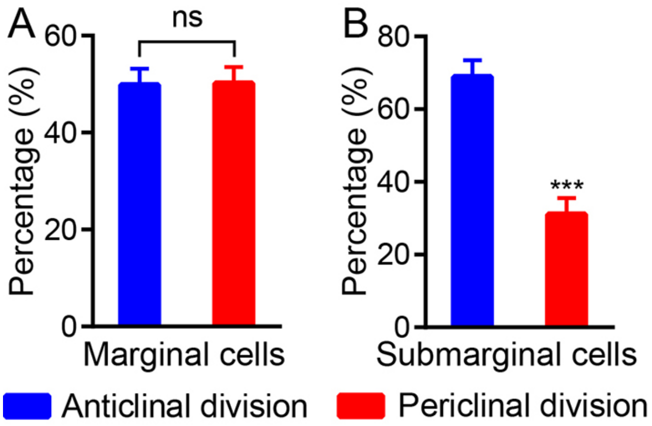

Figure S14. Quantification of cell division patterns in the marginal (A) and submarginal cells (B) that divided during the analyzed time period. The marginal cells and submarginal cells are defined as shown in Figure 6A-C. For cell division occurring from 0h to 48h, the percentage of anticlinal (or periclinal) division in the marginal cells is calculated as Number of a (or p) from the marginal cells at 48h / Total number of cell divisions (a and p) in the marginal cells that divided during the 0-48h period, and the percentage of anticlinal (or periclinal) division in the submarginal cells is calculated as Number of a (or p) from the submarginal cells at 48h / Total number of cell divisions (a and p) in the submarginal cells that divided during the 0-48h period. For cell division occurring from 48h to 96h, percentage of anticlinal (or periclinal) division in the marginal cells is calculated as Number of a (or p) from the marginal cells at 96h / Total number of cell divisions (a and p) in the marginal cells that divided during the 48-96h period; and percentage of anticlinal (or periclinal) division in the submarginal cells is calculated as Number of a (or p) from the submarginal cells at 96h / Total number of cell divisions (a and p) in the submarginal cells that divided during the 48-96h period. Bars: mean $\pm$ SE (n= 76 sets of time-lapse experiments with 50 independent samples in total). Each set of time-lapse experiments was quantified as the representative images shown in Figure 6A-C. (A) ns, $P > 0.05$, (B) ***$P<0.001$ (Student's two-tailed t-test). The source data for Figure S14 are included in Table S11. The same sets of time-lapse experiments were included for the quantification of cell division patterns shown in Figure S14 and Figure 6D. The number of total marginal and submarginal cells was used in the quantification shown in Figure 6D and Table S10, and the number of total cell division events during the analyzed period was used in the quantification shown in Figure S14 and Table S11.

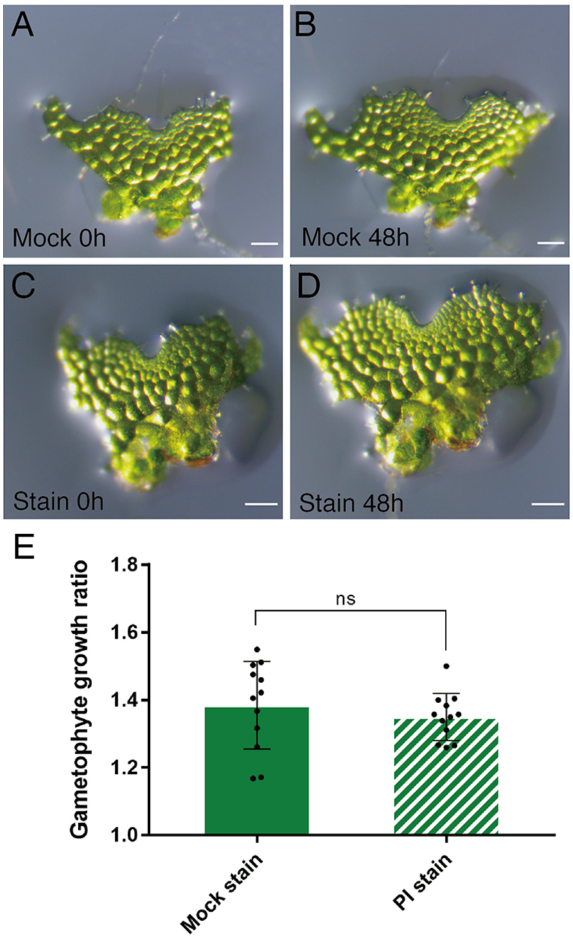

Figure S15. The growth rate of *W. obtusa* gametophytes over 48 hours after the Mock or PI stain. (A-B) Images of one representative *W. obtusa* gametophyte at 25 DAI at 0h (A) and 48h (B) after the Mock (water) stain. 12 independent gametophyte samples were included in the Mock stain. (C-D) Images of one representative *W. obtusa* gametophyte at 25 DAI at 0h (C) and 48h (D) after the PI stain. 12 independent gametophyte samples were included in the PI stain. All the mock-stained or PI-stained gametophyte samples were grown under the identical conditions and imaged at the same time. Scale bar: 100 µm. (E) The averaged growth rate of gametophytes over 48-h period after the Mock or PI stain. Growth rate of each individual gametophyte over 48 hours is calculated as the gametophyte area (at 48h) / the gametophyte area (at 0h). Bars: mean $\pm$ SE (n =12). ns, $P > 0.05$ (Student's two tailed t-test).

Table S1. Area quantification of each segmented cell from the *W. obtusa* gametophyte shown in Figure S3A-C and Figure 1U.

Table S2. Area quantification of each segmented cell from the *W. obtusa* gametophyte shown in Figure S3D-F and Figure 1W.

Table S3. The source data for Figure 4S.

Table S4. The source data for Figure 4T.

Table S5. The source data for Figure 4U.

Table S6. The source data for Figure 4V.

Table S7. The source data for Figure 4W.

Table S8. The source data for Figure 4X.

Table S9. Summary of percentage of four types of division in 77 three-celled packets during the proliferation of multicellular meristems in *Woodsia obtusa* gametophytes.

Table S10. Summary of cell division patterns in all the marginal cells and submarginal cells of *Woodsia obtusa* gametophytes.

Table S11. Summary of cell division patterns in the marginal cells and submarginal cells that divided during the analyzed period in *Woodsia obtusa* gametophytes.
